# Building a model: developing genomic resources for common milkweed (*Asclepias syriaca*) with low coverage genome sequencing

**DOI:** 10.1186/1471-2164-12-211

**Published:** 2011-05-04

**Authors:** Shannon CK Straub, Mark Fishbein, Tatyana Livshultz, Zachary Foster, Matthew Parks, Kevin Weitemier, Richard C Cronn, Aaron Liston

**Affiliations:** 1 Department of Botany and Plant Pathology, Oregon State University, 2082 Cordley Hall, Corvallis, Oregon 97331, USA; 2 Department of Botany, Oklahoma State University, 104 Life Sciences East, Stillwater, Oklahoma 74078, USA; 3 The Academy of Natural Sciences, 1900 Benjamin Franklin Parkway, Philadelphia, Pennsylvania 19103, USA; 4 Pacific Northwest Research Station, USDA Forest Service, 3200 SW Jefferson Way, Corvallis, Oregon 97331, USA

## Abstract

**Background:**

Milkweeds (*Asclepias *L.) have been extensively investigated in diverse areas of evolutionary biology and ecology; however, there are few genetic resources available to facilitate and compliment these studies. This study explored how low coverage genome sequencing of the common milkweed (*Asclepias syriaca *L.) could be useful in characterizing the genome of a plant without prior genomic information and for development of genomic resources as a step toward further developing *A. syriaca *as a model in ecology and evolution.

**Results:**

A 0.5× genome of *A. syriaca *was produced using Illumina sequencing. A virtually complete chloroplast genome of 158,598 bp was assembled, revealing few repeats and loss of three genes: *accD, clpP*, and *ycf1*. A nearly complete rDNA cistron (18S-5.8S-26S; 7,541 bp) and 5S rDNA (120 bp) sequence were obtained. Assessment of polymorphism revealed that the rDNA cistron and 5S rDNA had 0.3% and 26.7% polymorphic sites, respectively. A partial mitochondrial genome sequence (130,764 bp), with identical gene content to tobacco, was also assembled. An initial characterization of repeat content indicated that Ty1/*copia*-like retroelements are the most common repeat type in the milkweed genome. At least one *A. syriaca *microread hit 88% of *Catharanthus roseus *(Apocynaceae) unigenes (median coverage of 0.29×) and 66% of single copy orthologs (COSII) in asterids (median coverage of 0.14×). From this partial characterization of the *A. syriaca *genome, markers for population genetics (microsatellites) and phylogenetics (low-copy nuclear genes) studies were developed.

**Conclusions:**

The results highlight the promise of next generation sequencing for development of genomic resources for any organism. Low coverage genome sequencing allows characterization of the high copy fraction of the genome and exploration of the low copy fraction of the genome, which facilitate the development of molecular tools for further study of a target species and its relatives. This study represents a first step in the development of a community resource for further study of plant-insect co-evolution, anti-herbivore defense, floral developmental genetics, reproductive biology, chemical evolution, population genetics, and comparative genomics using milkweeds, and *A. syriaca *in particular, as ecological and evolutionary models.

## Background

The advent of next generation sequencing technology has presented the opportunity to obtain genome-scale data for any organism [1-3]. Detailed information about the characteristics of the genome of a non-model organism, especially the high-copy fraction, can be obtained even with very low overall coverage of a genome [4-6]. Repeat types can be characterized to assess the presence and prevalence of retrotransposons, DNA transposons, and other simple and low-complexity repeats [e.g., [4,7,8]]. Nearly complete sequences of organellar genomes are also readily obtained [e.g., [4,9-14]]. If genomic resources are available for a close relative, a portion of the gene space of the nuclear genome might also be surveyed [12]. In contrast to the above studies, next generation sequencing in non-model organisms has thus far primarily been used for characterization of transcriptomes [3] and as a tool for marker development in population and conservation genetics [e.g., [15,16]], phylogenetics [10,17], and other areas of evolutionary biology [e.g., [18]].

As the most intensively studied member of a genus that has been investigated extensively in diverse areas of evolutionary biology and ecology [e.g., [19-24]], the common milkweed (*Asclepias syriaca *L., Apocynaceae) is an attractive non-model target for the development of genomic resources. Interest in the genus has been focused on complex and specialized floral structures, particularly as they relate to insect pollination [21], and on the remarkable chemical ecology exemplified by cardenolide-sequestering specialist herbivores, such as the monarch butterfly (*Danaus plexippus*), that utilize *Asclepias *defense compounds to defend themselves against their own predators [25]. In the context of floral biology and plant-insect coevolution, *Asclepias *has the potential to add substantially to existing ecological and evolutionary model systems (e.g., *Arabidopsis, Mimulus, Aquilegia*), although genomic resources for *Asclepias *are currently far more limiting than for these model systems. Genomic resources have been developed for only two other species in the same order (Gentianales) as *Asclepias*: coffee (*Coffea; *http://www.coffeegenome.org) and Madagascar periwinkle (*Catharanthus roseus*: Apocynaceae), for which there are expressed sequence tag (EST) collections [26-28].

The aims of this study were to demonstrate how a small amount of Illumina short read sequence data could be useful for whole genome characterization of a plant species with no prior genomic information and to develop genomic resources for milkweeds. To these ends, the genome of *Asclepias syriaca *was characterized using 0.5× sequence coverage obtained from a single lane of 40 bp Illumina reads. Reference-guided assembly was used to assemble and characterize the high copy fraction of the *A. syriaca *genome, including the complete chloroplast and partial mitochondrial genomes and a nearly complete nuclear ribosomal DNA cistron and 5S rDNA sequence. *De novo *assemblies were used to characterize the repeat structure of the genome and to develop nuclear markers consisting of low-copy nuclear loci for phylogenetics and microsatellite loci for population genetics. This study highlights the extent to which a non-model plant genome can be characterized with low coverage genome sequencing and represents the first step in developing the resources needed to develop *Asclepias *as a new genetic model system in ecological and evolutionary studies [29].

## Methods

### Genome size estimation

Leaves were collected from five *A. syriaca *individuals from an experimental agricultural population in western Illinois and frozen at -20°C. Samples of approximately 3×3 mm green leaf tissue were chopped in 150 μl Partec CysStain UV Precise P Nuclei extraction buffer (made 1.0% w/v PVP-40; Partec North America, Inc., Swedesboro, NJ, USA) on ice in the presence of (~8×8 mm) B-73 maize (1C = 2.725 pg) leaf tissue as internal reference. Upon filtering and addition of 600 μl CysStain UV Precise P Staining Buffer, samples were run on a Partec PA flow cytometer until at least 1000 counts were recorded for both the sample and reference peaks. The estimated DNA content values for the five individuals were averaged and the genome size calculated using a standard conversion factor [30].

### Illumina library preparation and sequencing

Leaf tissue was sampled from a single individual from a natural population of *A. syriaca *in Ogle County, Illinois [*Fishbein 4885 *(OKLA)]. Total genomic DNA was extracted using the Wizard^® ^Genomic DNA Purification kit (Promega, Madison, WI, USA) following homogenization of 100 mg of silica-dried tissue flash-frozen in liquid N_2 _and ground with mortar and pestle. A BioRuptor Sonicator (Diagenode Inc., Denville, NJ, USA) was used to shear 2.5 μg of the extracted DNA using 15 one min cycles (30 sec on/30 sec off, setting 'high', 4°C). Subsequent library preparation followed the standard multi-step Illumina protocol [31]. Ligated, size-selected fragments (~280 - 320 bp) were amplified through 18 cycles of polymerase chain reaction (PCR), using Phusion High-Fidelity PCR Master Mix (New England Biolabs, Ipswich, MA, USA) and standard Illumina primers. Resulting product concentrations were determined using a Nanodrop 1000 (ThermoFisher Scientific, Wilmington, DE, USA).

Enriched, cleaned product was diluted to 5 pM and submitted for sequencing on an Illumina GAII sequencer at the Center for Genome Research and Biocomputing (CGRB) at Oregon State University (Corvallis, OR, USA). Denaturation, cluster generation, and subsequent sequencing followed the manufacturer's recommendations. Sequencing was completed over 40 cycles on a single lane of the Illumina GAII. Image analysis, base-calling and error estimation were performed using the Illumina GA Pipeline version 1.5. Images were collected from 120 tiles, averaging over 107,000 clusters/tile, and resulted in over 420 Mbp of sequence data after quality filtering.

### Reference-guided assembly

Reference-guided assembly of microreads was facilitated using a pipeline of five open source scripts called "alignreads." The pipeline incorporates: YASRA [32], a short read assembler; NUCMER, an aligner from the MUMmer 3.0 suite [33,34]; delta-filter, a utility program from the MUMmer 3.0 suite that removes excessive/erroneous alignments; "sumqual," a python script created to integrate the NUCMER alignment information with the YASRA assembly information; and "qualtofa," another python script that reformats and filters the output of sumqual based on user inputs. A consensus sequence of the aligned contigs is produced, which can be masked based on base call proportion and coverage depth; single nucleotide polymorphisms (SNPs) can be masked similarly using separate user specifications. Scripts are available for download at http://milkweedgenome.org. Read depth was determined by calculating median base sequencing depth from the alignreads output.

#### Chloroplast Genome

The reference-guided assembly of the chloroplast genome was completed in alignreads using the chloroplast genome sequence of oleander (*Nerium oleander*: Apocynaceae; M. Moore and D. Soltis, unpublished data; see [17] for coding sequences) with only one copy of the inverted repeat included as the reference and no masking. A second alignment of the YASRA contigs was completed using Mulan [35], and the resulting consensus sequence was compared to the alignreads output and tested as an alternate reference input for alignreads. All of the consensus sequences were aligned to each other and the oleander reference using MAFFT 6.240 [36]. A preliminary annotation of the *A. syriaca *chloroplast genome was completed using DOGMA [37]. The annotations of protein coding regions, ribosomal DNA (rDNA), and sequences corresponding to transfer RNAs (tRNAs) were improved by fixing annotation and assembly mistakes (e.g., no start or stop codon, internal stop codons, frameshifts, endpoints of tRNA sequences) through examination of the actual assembly of the short reads at that location in Tablet vers. 1.10.09.20 [38] and/or comparisons to ortholog sequences from other asterids obtained from NCBI GenBank (e.g., *Nerium, Coffea*, *Solanum*). Various versions of the chloroplast genome sequence were used as a reference in alignreads to further refine the sequence based on new assemblies. Discrepancies between the assembly consensus sequences in introns and intergenic spacers were explored at the microread-level in Tablet to identify the correct sequence. An *Asclepias tuberosa *sequence [GenBank:GQ248251.1] served as a reference in alignreads to correctly assemble the *trnH *- *psbA *region.

In order to produce the highest quality *A. syriaca *chloroplast genome sequence and annotation, the consensus sequences were also aligned to chloroplast genome sequences for other individuals of *A. syriaca *produced in alignreads and Velvet 1.0.12 [39] based on either 80 bp single end or paired end Illumina sequences (S. Straub and A. Liston, unpublished data). Discrepancies between the consensus sequences from these assemblies and the original consensus sequences were resolved as either intraspecific variation, assembly or alignment errors in the 40 bp read data set, or unresolved assembly errors. Informatically unresolved errors (e.g., AT-rich repeats) were corrected by obtaining Sanger sequences spanning those regions. Gene sequences that were expected to be divergent from the reference (e.g., *ycf1*) and putative pseudogenes were also checked by Sanger sequencing. PCR reactions contained ~10-20 ng template DNA and had a final concentration of 1× Phusion^® ^Flash High-Fidelity PCR Master Mix (Finnzymes) and 0.2 μM of primer. PCR cycling conditions were 98°C for 30 s followed by 25 cycles of 98°C for 10 s, primer set specific annealing temperature for 30 s, 72°C for 30 s, and a final extension of 72°C for 5 min. See Table 1 for primer sequences and annealing temperatures. Cycling extension times were increased to 60 s and final extension times to 10 min for *accD *and *clpP*. Cycling extension times were increased to 90 s and final extension times to 10 min for *ycf1-ndhF *and *rps15-ycf1*. The success of PCR reactions was confirmed using agarose gel electrophoresis. The chloroplast and mitochondrial paralogs of *accD *were co-amplified, bands excised from a 1.2% agarose gel, and purified using a QIAquick Gel Extraction Kit (Qiagen, Inc.). Due to low yield, the mitochondrial product was re-amplified using 1 μl of extract as PCR template prior to sequencing. For additional chloroplast *accD *sequencing, the mitochondrial product was virtually eliminated from the PCR pool by raising the annealing temperature to 61°C. Sequences were produced using standard dye termination techniques by High-Throughput Sequencing Solutions at the University of Washington http://www.htseq.org or the Oregon State University CGRB. The quality of the finished *A. syriaca *chloroplast genome sequence was assessed by using it as the reference sequence in alignreads to re-assemble the 40 bp reads and any discrepancies were explored and corrected.

**Table 1:** Primers used for Sanger sequence finishing of the *Asclepias syriaca *chloroplast genome

Region spanned	Orientation	Primer Sequence (5'-3')	Annealing Temp. (°C)
*ndhC-trnV*	FORWARD	GCTCGATTCGAATTGTCAAGTCATCC	57
	REVERSE	AGGCCCTCCGATAGGATACTCAAA	

*trnQ-psbK*	FORWARD	AAACCCGTTGCCTTACCACTTG	57
	REVERSE	CACGGGTATGACTGGCATAACATC	

*rbcL-accD*	FORWARD	ATCCGTGAGGCTAGCAAATGGAGT	59
	REVERSE	AGGCCCTAGTCCACCAATATGGAA	

*accD*	FORWARD	TAATAAACAATGCTAGCGATTGGG	57, 61
	REVERSE	TACAATTAGATCTTAGGTCGCCAA	
	FORWARD	GAACCTACACGCAAGCTAGTG	
	FORWARD	TAAATGGCATTCCCATAGCAGT	
	FORWARD	TGATATCGATGCGCAAGCTAG	
	REVERSE	CCTATAAATGTAACTAGCACTGCA	
	REVERSE	GGTAGCGTATTCAATCAAACGG	
	REVERSE	CCAAGTATCCGGATCGATCGA	

*clpP*	FORWARD	AATTGTATACATAATGGGCTGGT	57
	REVERSE	TATTTATTCATTGCCCTGTTCGT	
	FORWARD	AACTACTATGATGGCTCCGTTG	
	FORWARD	CATAAGACCCATAATTTGATTTGAG	
	FORWARD	GTTTATGCTGTACTCCGGGTA	
	REVERSE	GGTCAATTTTATTGTAAAGCCGTAT	
	REVERSE	TATGCAATTTATAAAACCAGATGTCC	
	REVERSE	GTGAATTGAAAAAGTAGAACGTCAT	

*rps8 -rpl14*	FORWARD	CTCAGCAATAGTGTCTCTGCCCAT	57
	REVERSE	AGAGCTGTAATTGTGCGTACCCGT	

*ycf1-ndhF*	FORWARD	TCCATCCATATCCCAATTCCATTCA	55
	REVERSE	GGGCATTAGAGGATTGGCTAAAGT	
	FORWARD	ACGCCTCTGCATCTAGTATTG	
	REVERSE	GGATCTTTTGTCGAGAGCTTC	

*ndhG-ndhI*	FORWARD	ATGCGGGCGGGTTGATATATGGTT	59
	REVERSE	ATTGCTTTGGGTCGGTTACCAACGTC	

*rps15-ycf1*	FORWARD	GGCCTAATTGATACCTCAGCGGTT	58
	REVERSE	TTATCAACGATGGAACCGCCCACT	
	FORWARD	TATGTCTCGGTCTAGGAGAGG	
	REVERSE	TGTACAAGTTGATTCAGGCGA	

For each region, additional primers appearing after the two original PCR primers are internal sequencing primers.

The final annotation of the *A. syriaca *chloroplast genome was accomplished using BioEdit 7.0.5.3 [40] and visualized using GenomeVx [41]. The repeat content of the chloroplast genome was determined using the NCBI BLAST tool blast2seq. Only repeat motifs of greater than 30 bp and 85% sequence identity were characterized. Sequence identities were calculated in BioEdit on sequence alignments produced in MAFFT with the gapped positions removed. Ka/Ks values for pseudogenes were calculated using the Bergen Center for Computational Science Ka/Ks calculation tool http://services.cbu.uib.no/tools/kaks.

#### Mitochondrial Genome

The mitochondrial genome of *A. syriaca *was assembled using the alignreads pipeline and the 430,597 bp mitochondrial genome sequence of *Nicotiana tabacum *[GenBank:BA000042.1] [42] as a reference. Mitofy [43] was used to characterize the complement of protein coding, rRNA, and tRNA genes in the resulting contigs. The annotation of the mitochondrial genome of *N. tabacum *was updated using Mitofy before comparison of the gene content of the two genomes. BLAT [44] under default parameters was used to estimate the percentage of each *A. syriaca *mitochondrial gene assembled by calculating the proportion of the total length of the *N. tabacum *mitochondrial genes with hits in the *A. syriaca *contigs. The presence or absence of the *cox1 *group I intron in *Asclepias *was determined by using the *Hoya sikkimensis *(Apocynaceae) *cox1 *sequence [GenBank:AJ247588.1] as a BLAT reference.

#### Ribosomal DNA

The nuclear rDNA cistron, including 18S, 5.8S, and 26S, was assembled in alignreads using a 669 bp partial sequence from *A. syriaca *[GenBank:AM396892] spanning the internal transcribed spacers (ITS) as the reference. Following the initial assembly, the assembly was repeated using the reverse complement of the reference sequence as the new reference to correct for an inconsistency in YASRA that caused the assembly result to be strand specific. A consensus sequence of the contigs obtained through the first two analyses was then used as the reference for a final assembly. A 5S rDNA sequence for *A. syriaca *was assembled using a 330 bp sequence including the 5S locus from *Solanum iopetalum *[GenBank:AJ226045] as a reference in both the forward and reverse directions.

To assess intragenomic polymorphism, the complete genomic read pool was filtered for reads matching the consensus sequences of the assembled rDNA loci using BLAT and custom scripts [45]. Reads were discarded if their average Phred score was less than 20, and the remaining reads filtered so that any base in a read with a Phred score less than 20 was converted to 'N' using the FASTX-Toolkit [46]. Modified reads were assembled against the reference sequence using BWA [47], and at each position the number of reads differing from the consensus was tallied and a proportion calculated (script available from KW). In order to estimate the error introduced by the sequencing method, the same procedure was applied to the PhiX174 control lane of the sequencing run.

Positions with 2% or more of reads differing from the consensus were considered to represent true polymorphism. This cutoff is comparable to error rates previously found using a similar quality-filtering scheme [48], but may be somewhat conservative as it is much higher than the error seen in the PhiX lane. See additional file 1: Polymorphism detected among Illumina reads for the PhiX sequencing control. All positions of the PhiX genome exhibited <0.7% differing reads except position 3132, which had >17% of reads differing from the consensus. While the reason for this anomaly is uncertain, the overall average error rate in the PhiX lane is <0.05%.

#### *De novo *assembly

##### Data Quality Control

Perl scripts were used to remove microreads containing N's and those reads corresponding to Illumina adapter sequences from the chastity-filtered short read pool to create a cleaned read pool [45]. The remaining reads were trimmed of bases of low quality or unscorable quality (quality score "B" in Illumina fastq files) and all bases subsequent to these bases to produce a cleaned and trimmed read pool [45].

*De novo *assembly of the cleaned read pool was performed using VelvetOptimiser 2.1.7 [49] and Velvet 1.0.12 with the minimum contig length set to 100 bp. Hash lengths between 19 and 31 were screened to find the optimal hash length for maximizing the total bases included in contigs. The expected coverage and the optimum coverage cutoff were estimated by VelvetOptimiser to be 2 and 0.3777216 respectively. This analysis was repeated for the cleaned and trimmed read pool. Use of the cleaned read pool resulted in more total bases in contigs; therefore this pool was used for all downstream *de novo *analyses. In order to further characterize the *A. syriaca *nuclear genome, BLAT was used to remove reads corresponding to nuclear rDNA and the chloroplast and mitochondrial genomes using the sequences generated through reference guided assembly. The *de novo *assembly was repeated to produce the set of contigs used for the non-rDNA repetitive element categorization and microsatellite marker development. The organellar genome and rDNA BLAT analyses were also used to estimate the percentage of overall reads corresponding to the high copy fraction of the genome.

##### Repetitive Element Characterization

Non-ribosomal nuclear repetitive elements were characterized by submitting the *de novo *contigs to the MAKER [50] web annotation service http://www.yandell-lab.org/software/mwas.html and categorizing the RepeatMasker [51] output from the MAKER run. The percentage of unique read hits to these contigs was determined using BLAT, with minimum sequence identity set to 70%, to find hits to the contigs of putative DNA transposon or retrotransposon origin. The overall percentage of reads corresponding to known repetitive elements was calculated using BLAT (minimum sequence identity = 70%) to determine the number of reads that hit the Viridiplantae repeat sequences downloaded from the Repbase Update release 15.07 database [52].

##### Microsatellite Marker Development

*De novo *contigs of at least 150 bp, to allow for sufficient flanking sequence for primer design, were scanned for microsatellites using BatchPrimer3 [53]. Primers were designed flanking perfect di- and trinucleotide repeats of four or more repeat units and perfect tetra-, penta-, and hexanucleotide repeats of three or more repeat units such that the PCR amplicon sizes ranged between 100 and 450 bp and primers met the default criteria for SSRs primers (Table 2; also see additional file 2: Primer sets designed using BatchPrimer3 for 184 nuclear microsatellite loci in *Asclepias syriaca *not tested for amplification success). Contigs containing at least one microsatellite locus for which primers could be designed were submitted to the PLAN web service [54], with the 'DNA to DNA: blastn against NCBI NT 20081206 database, top 10 hits with e < = 0.1 cut-off' option selected, in order to check if any chloroplast or mitochondrial sequences had not been eliminated from the read pool used to generate the *de novo *contigs. To produce the best set of primers for candidate nuclear microsatellite locus development, those primer sets originating from contigs with a chloroplast or mitochondrial BLAST [55] hit with an E-value of 1e^-5 ^or lower were removed from the candidate pool.

**Table 2:** Primers, PCR reaction conditions, and characteristics of microsatellite loci tested for amplification success in *Asclepias syriaca*

Primer Set Name	Orientation	Primer Sequence (5'-3')	Annealing Temp. (°C)	Repeat Motif	Product Size (bp)
As104467	FORWARD	ACTTTTTGTTCTGTTCCGAGT	52.9	TA_6_	100
	REVERSE	CGGGGCTAGTATAGAAGAAGA			

As106994	FORWARD	AGGAAGCCAATTCAAGTAAAG	48.9	GA_5_	129
	REVERSE	GAAAATCCTTGCATGAAACT			

As12122	FORWARD	TTGGTTGTTGGAACTTACACT	50.4	ATTC_4_	115
	REVERSE	AAGCACAAGAATCAGAACAAT			

As125636	FORWARD	GAACTCCTATGATCTTTCATTT	50.8	TC_4_...TA_4_*	136
	REVERSE	AGACATCATGAACCCTCTTTA			

As12641	FORWARD	CTCTTCATCTGGTTTCTCTTG	49.0	TA_4_	130
	REVERSE	TGCATACAAATTTAGGTGATTC			

As13110	FORWARD	AACCATAAATGCAGGTTCTTT	50.9	TA_4_	101
	REVERSE	GCATATCTAGCTCGCATTTAG			

As16135	FORWARD	TTAGGTTGTGAGAAGGGATTT	50.3	CCT_4_	100
	REVERSE	GCTTTTATTAATGTGGGAGGA			

As16369	FORWARD	AACGCTTATCCCTCACTTTAC	51.5	TA_5_	100
	REVERSE	CCGAACTCTATATGAGTCCAAT			

As196582	FORWARD	GCATTTATTGCATTGTCAAA	50.5	TGA_5_	113
	REVERSE	GCCGTTGTTCCTCTATAATTT			

As2003a	FORWARD	CCCTCGTTATTGAGAAAATAGA	50.5	ATA_5_	105
	REVERSE	GGCTCTTACCAAAGAAAAGAT			

As20679a	FORWARD	GTCATCGGAAGCTCTTTTC	52.7	CTT_4_	104
	REVERSE	AGTCAAAAGTCACACTCATCTG			

As2338	FORWARD	CATCTTGGTTAGCTCGTAAAC	51.7	TC_5_	104
	REVERSE	CCTTACCTAGGATGAAAGGAG			

As23491a	FORWARD	ATTTCCTCCATTTGTTCTTTC	50.0	TC_5_	101
	REVERSE	GAAGTTTGTCTGAAATGGTTG			

As2366	FORWARD	TTGGTGTTGTAAAGTGTTCCT	54.5	TA_5_	101
	REVERSE	ACCAGCTCGTCAGTCAAG			

As23942	FORWARD	GTGATTTCCATTTTACCATACC	51.7	AG_5_	100
	REVERSE	ATCCTTAGCAAATAGGAGTCG			

As23961	FORWARD	ACTCTTGTTATTTTGGGGAAT	49.3	TG_5_	108
	REVERSE	CAAAAGCTTTACACGATCAAT			

As282930	FORWARD	AACTTAGCTTTGGATGATGTTT	50.1	TAA_4_	102
	REVERSE	CAATTGGGCTTGTATATCAGA			

As28460	FORWARD	CACAAGATGAACATCAAAACC	49.6	AT_5_	100
	REVERSE	TTGAGTATATTTGTTCCCCAAT			

As292191	FORWARD	GGAGGGTCTAATGTCTGATGT	51.3	TG_5_	125
	REVERSE	ATTCACACATCCCATACCAT			

As43163	FORWARD	TATTCCAAGAAATTGGTCAAC	49.7	TTA_4_	110
	REVERSE	CATGGAAGAAGAACAAAACAG			

As5768	FORWARD	AAGGGAAATGATCCTTAGGTA	51.0	AG_6_	108
	REVERSE	TTCGAAAGAGTTCCACATCTA			

As71725	FORWARD	TTTACGACTCATGTACTCTTCC	50.8	AG_4_...TG_5_*	133
	REVERSE	GTAATACCGGAAAATGACCTC			

As77693	FORWARD	GTCAGTCAACTCAACAATGCT	51.0	TC_6_	102
	REVERSE	TCCCAAAACTCAGATCTAACA			

As81784	FORWARD	AATGTAAAGGGAGATGTTTATCA	52.0	TAT_4_	129
	REVERSE	AGCATCAAGAACTTGAACAGA			

As99212	FORWARD	TCTATGGTCAACAATTTCTGC	53.4	AT_5_	153
	REVERSE	TTTATAGGGGTAGCGTAGGTG			

*Repeat motifs are separated by 27 bp for primer set As71725 and 46 bp for primer set As125636.

Primers were tested for amplification success for the 25 top candidate loci, which were selected for having the highest number of repeat units among all candidates. PCR amplification was conducted on genomic DNA of the same *A. syriaca *accession used for genome sequencing in 50 μl reactions. Each reaction consisted of 1 μl DNA extract template, 1.25 U GoTaq^® ^Flexi DNA Polymerase and 1× Green Flexi Reaction Buffer (Promega, Madison, WI, USA), 2.5 mM MgCl_2_, 200 μM dNTPs, and 0.8 mM amplification primers. PCR was carried out in a C1000™ thermal cycler (BioRad, Hercules, CA, USA) under the following conditions: initial denaturing at 95°C for 2 min, 35 cycles of 95°C for 30 sec-variable annealing temperature for 30 sec-72°C for 1 min, followed by a final extension at 72°C for 5 min. See Table 2 for annealing temperatures. Amplicons of the predicted size were confirmed using agarose gel electrophoresis.

### Nuclear genome characterization and development of low copy nuclear markers

In order to provide an initial characterization of nuclear gene content and coverage, all 19,899 available ESTs for *Catharanthus roseus *were downloaded from GenBank on 29 November 2010. See additional file 3: List of GI numbers for 19,899 *Catharanthus roseus *ESTs downloaded from GenBank and assembled into unigenes. The sequences were subjected to simple cleaning in SeqClean (Dana Farber Cancer Institute; available at http://sourceforge.net/projects/seqclean). Cleaned and trimmed sequences were assembled into unigenes using iAssembler v1.2 (available at http://bioinfo.bti.cornell.edu/tool/iAssembler). Unigenes with BLAT hits corresponding to the rDNA or chloroplast or mitochondrial genomes of *A. syriaca *were excluded from this set. Simple repeats, low complexity sequences, and long terminal repeats were masked in the remaining unigenes using the RepeatMasker web service [[51]; available at http://www.repeatmasker.org/cgi-bin/WEBRepeatMasker]. BLAT with a tile size of 7 and minimum sequence identity of 80% was used to find read hits to the remaining putatively nuclear masked unigenes. The number of unique microread hits per unigene was used to calculate the hits per kb of sequence based on the length of gene. Coverage for these genes was then estimated using the formula [(hits per kb of sequence × length of the microread)/1000]. Coverage is overestimated when reads overlap. JMP 9.0.0 (SAS Institute, Inc.) was used to calculate coverage distributions. A Monte Carlo resampler implemented in PopTools [56] was used to calculate 95% bootstrap confidence intervals (1,000 iterations) for median hits per kb and coverage values.

With low genome coverage, few if any single-copy nuclear genes were expected to have been assembled in *de novo *contigs. In order to extract useful information for nuclear marker development from the unassembled, uncleaned pool of reads and further explore the *A. syriaca *gene space, BLAT with a tile size of 7 and minimum sequence identity of 80% was used to find reads that hit alignments for 1,086 single-copy orthologs from other asterids [e.g., tomato (*Solanum lycopersicum*), potato (*Solanum tuberosum*), pepper (*Capsicum annuum*), coffee (*Coffea canephora*)] and *Arabidopsis thaliana *[COS II markers; [57]]. The average length of sequences included in each alignment was used as the gene length in subsequent calculations, which were completed as described above for the *Catharanthus *unigene set.

The COSII pool of candidate genes was most appropriate for the development of markers intended for phylogenetics because single-copy genes are preferred in order to avoid ortholog/paralog conflation. MAFFT 6.240 was used to align the *Asclepias *microreads with the COSII alignments using the localpair option and 1,000 cycles of iterative refinement. Primers were designed in exons flanking the putative locations of one or more introns based on the location of introns in *A. thaliana *indicated by the Sol Genomics Network's intron finder application http://solgenomics.net/tools/intron_detection/find_introns.pl for genes with 0.5× or greater coverage (Table 3). If possible, primers were designed with GC clamps and were chosen from sections of the read identical to or highly similar to the coffee sequence without mismatches near the 3' end of the primer sequence.

**Table 3:** Primers designed from *Asclepias syriaca *microreads hitting COSII alignments

Primer Set Name	Orientation	Primer Sequence (5'-3')	Annealing Temp. (°C)
At1g24360a	FORWARD	GCAAGATCATCAAAGGAGGCA	59.0
	REVERSE	GATTTAGATCAATAACCTCCTGCCA	

At1g24360b	FORWARD	GGAGGTTATTGATCTAAATCTCAC	56.3
	REVERSE	AACAAGGCCAACAACTGATG	

At1g24360c	FORWARD	CAATTACAGTGCTGCAAAAGCTG	60.0
	REVERSE	CATATCAGATGCAATGAATCCGGG	

At1g44760a	FORWARD	TACTCATGTTACTAATAAGGGTGA	-
	REVERSE	CTTTGCTTTGCTTCCTCACTC	

At1g55880a	FORWARD	ATGTACACAAAAGAGGAGGCTG	54.0
	REVERSE	GATGCCTCATCCCACTATCAC	

At1g77370a	FORWARD	CCAAAGCGCCATCTACTCCA	60.0
	REVERSE	GGCCAACCAAATCCAGAAGAAT	

At2g03120a	FORWARD	GAAGGTAGATCCAAATTTGAATGTC	58.0
	REVERSE	GCAAGTCAACACAGCATTAAC	

At2g06530a	FORWARD	GTGAAGGTAATGGCCAAAGATC	58.0
	REVERSE	GTAACACCTTTCATTGCTTCTCC	

At2g21960a	FORWARD	ATTGGGGTTTCTTTGTTCCATAC	53.0
	REVERSE	GCAGATTGGCCAACAACTTTG	

At2g21960b	FORWARD	GTTGTTGGCCAATCTGCAGA	59.0
	REVERSE	TTTGTTGCTGATCTTTGCTAAGC	

At2g30200a	FORWARD	ACAAGCTGTTGGAATGGGTG	55.0
	REVERSE	ATCTCAACTGCAGCTAAGCTG	

At2g30200b	FORWARD	ATCTATGTCACCAGCTTAGCTG	58.3
	REVERSE	TTTGAATGATTTTGCCTTTGCTTC	

At2g30200c	FORWARD	CAAAGGCAAAATCATTCAAAGC	56.3
	REVERSE	TGCATCAACATTTGATATTACTGG	

At2g34620a	FORWARD	TTCAGAAAGGTCATCAACAAATG	57.8
	REVERSE	GCTGAAAGTGAACAAACCTGG	

At2g34620b	FORWARD	TGCCAACATTTCTTTACAATTCAAG	52.0
	REVERSE	none	

At2g47390a	FORWARD	GTCTTGGTACAAAACACGCAAG	59.0
	REVERSE	TCAGTTTTAGATTCTTTAGAGGTAAG	

At2g47390b	FORWARD	CTAATGAGTTTGCTGGCATTGG	-
	REVERSE	CACCATGTCCTTTGAGTGCG	

At4g13430a	FORWARD	GAGCAAAACATAAAGTACTTCTA	48.0
	REVERSE	CATTTCACCATCCATAACAA	

At4g13430b	FORWARD	GTGCCTCCAACATTGAGATTTG	59.0
	REVERSE	CATTCTCTAGCCAAAGCACGA	

At4g13430c	FORWARD	GTGCTTTGGCTAGAGAATGCA	60.0
	REVERSE	CAAGACAAGCACCACAACTAGG	

At4g26750a	FORWARD	GCCTGATGACCATTTACATCTTG	-
	REVERSE	TACAGGTCTCCTCCCTTCTTTC	

At5g13420a	FORWARD	TGGTATGACAACCTGTGCCG	55.0
	REVERSE	GTATCATCAGCAAGTCTTGGTGAA	

At5g13420b	FORWARD	CTCCATGCGTCCCTTCAATC	60.5
	REVERSE	TGGCACCTTTCTTCACCAGAG	

At5g23540a	FORWARD	GTGTTGAAGCGGTTGATCATG	-
	REVERSE	TCCATCTGTCCATTTTTTCTTATGA	

At5g23540b	FORWARD	none	52.0
	REVERSE	AAGGCATCAATTACCACTTTCC	

At5g49970a	FORWARD	GTAGCTGCTCGTCATTTATATC	55.8
	REVERSE	GAGAATCCAAACATTGCATCA	

At5g49970b	FORWARD	TTGATGCAATGTTTGGATTCTC	57.8
	REVERSE	GGCACACAATTTTGGAGCAG	

Multiple primer sets were designed for loci where multiple read hits allowed potential amplification of different introns. Primer set names correspond to the orthologs of these genes in *Arabidopsis*. Listed annealing temperatures were used in amplifications of *Asclepias *samples. An annealing temperature of 55°C was used for all loci for amplifications of other Apocynaceae samples.

The primers designed using the *A. syriaca *microreads were tested for amplification success and their utility in phylogenetic studies of *Asclepias *and across Apocynaceae. Vouchers for all DNA samples used are listed in Table 4. DNA was extracted as described above for *A. syriaca*. PCR amplification was conducted in 50 μl reactions on genomic DNA of the same *A. syriaca *accession used for genome sequencing, 16 accessions of 13 other species of American *Asclepias*, including four accessions of two morphotypes of *A. pringlei*, one species of *Gomphocarpus *belonging to the sister clade to American *Asclepias*, and one sample each of successive sister groups to the clade including *Asclepias *and *Gomphocarpus *(*Calotropis *and *Pergularia*). Each reaction consisted of 1 μl template DNA extract, 1.25 U GoTaq^® ^Flexi DNA Polymerase and 1× Green Flexi Reaction Buffer (Promega, Madison, WI, USA), 2.5 mM MgCl_2_, 200 μM dNTPs, and 0.8 mM amplification primers. PCR was carried out in a C1000™ thermal cycler (BioRad, Hercules, CA, USA) under the following conditions: initial denaturing at 94°C for 2 min, 35 cycles of 94°C for 30 sec-variable annealing temperature for 30 sec-72°C for 1 min, followed by a final extension at 72°C for 5 min. See Table 3 for primer sequences and annealing temperatures.

**Table 4:** Herbarium voucher specimens for DNA samples included in the COSII marker screen

Species	Voucher	Provenance
*Alyxia grandis *P.I. Forst.	D. J. Middleton 703 [A]	Australia
*Alyxia oblongata *Domin.	D. J. Middleton 702 [A]	Australia
*Asclepias californica *Greene ssp. *californica*	Lynch 10,779 [LSUS]	USA. California. San Bernardino Co.
*Asclepias connivens *Baldwin ex Elliott	Lynch 12,350 [LSUS]	USA. Florida
*Asclepias coulteri *A. Gray	Fishbein 5172 [OKLA]	Mexico. Querértaro. Mpio. Landa
*Asclepias cryptoceras *S. Watson ssp. *davisii*	Fishbein 5723 [OKLA]	USA. Oregon. Crook Co.
*Asclepias fascicularis *Decne.	Fishbein 5886 [OKLA]	USA. California. Siskiyou Co.
*Asclepias glaucescens *Kunth	Lynch 14,142 [LSUS]	Mexico. Oaxaca
*Asclepias oenotheroides *Schltdl. & Cham.	Lynch 13,339 [LSUS]	USA. Texas
*Asclepias otarioides *E. Fourn.	Fishbein 5857 [OKLA]	Mexico. Distrito Federal. Del. Tlalpan
*Asclepias pringlei *(Greenm.) Woodson	Fishbein 5845 [OKLA]	Mexico. Zacatecas. Mpio. Tlaltenango
*Asclepias pringlei*	Ventura 2807 [ARIZ]	Mexico. Distrito Federal. Del. Milpa Alta
*Asclepias aff. pringlei*	Fishbein 5119 [OKLA]	Mexico. Michoacán. Mpio. Coalcomán
*Asclepias aff. pringlei*	Fishbein 5175 [OKLA]	Mexico. Querétaro. Mpio. Pinal de Amoles
*Asclepias sanjuanensis *K.D. Heil, J.M. Porter & S.L. Welsh	Ellison s.n. [OKLA]	USA. New Mexico. San Juan Co.
*Asclepias stenophylla *A. Gray	Fishbein 5394 [OKLA]	USA. Colorado. Boulder Co.
*Asclepias subulata *Decne.	Fishbein 3147 [WS]	Baja California Sur. Mpio. Comondú
*Asclepias viridis *Walter	Lynch 13,072 [LSUS]	USA. Texas
*Baissea multiflora *A. DC.	F. Billiet S3853 [BR]	Cultivated. Belgium. Nat. Bot. Gard. Belgium
*Calotropis procera *(Aiton) W.T. Aiton	Fishbein 5472 [OKLA]	Cultivated
*Epigynum auritum *(C.K. Schneid.) Tsiang & P.T. Li	D. J. Middleton et al. 1457 [A]	Thailand
*Epigynum cochinchinensis *(Pierre) D.J. Middleton	D. J. Middleton 209 [A]	Thailand
*Gomphocarpus cancellatus *(Burm. f.) Bruyns	Drewe 534 [K]	South Africa
*Marsdenia glabra *Constantin	D. J. Middleton et al. 1123 [A]	Thailand
*Oncinotis glabrata *Stapf ex Hiern	C. C. H. Jongkind et al. 1315 [US]	Ghana
*Oncinotis tenuiloba *Stapf	T. Abbott 7707 [Z]	South Africa
*Pergularia daemia *(Forssk.) Chiov.	Fishbein 5445 [OKLA]	Namibia
*Telosma cordata *(Burm. f.) Merr.	T. Livshultz 01-33 [BH]	Cultivated. USA. Cornell University

For the Apocynaceae-wide analyses, species from four tribes (Marsdenieae, Baisseeae, Apocyneae, Alyxieae) at increasing phylogenetic distance from *Asclepias *[58] were selected for primer testing. Pairs of species from three tribes were used: *Telosma cordata *and *Marsdenia glabra *(Marsdenieae), *Baissea multiflora *and *Oncinotis tenuiloba *(Baisseeae), *Epigynum auritum *and *E. cochinchinense *(Apocyneae); *Oncinotis glabrata *was substituted for *O. tenuiloba *for six of the primer pairs. In addition, the even more distantly related *Alyxia oblongata *and, for six of the primer pairs, *Al. grandis *(Alyxieae) were tested. DNA was extracted using a modified DNeasy method [58]. PCR reactions were performed in 30 μl reaction volume with Apex™ Taq DNA Polymerase Master Mix (Genesee Scientific) diluted to 1× with final concentrations of 1.5 mM MgCl_2 _and 0.5 mM forward and reverse primers (Table 3). One μl of unquantified DNA extract was added to each reaction. PCR was performed in an Eppendorf Mastercycler gradient thermocycler with an initial melting step of 94°C for 3 min, followed by 35 cycles of 94°C for 1 min, 55°C for 1 min, 72°C for 1 min, with a final extension of 72°C for 7 min. PCR products were examined for amplification success and product sizes estimated by agarose gel electrophoresis for both the *Asclepias*-specific and family-wide surveys.

## Results & Discussion

### Genome size estimation

The average 2C value for the five *A. syriaca *individuals was 1.68 pg (SD 0.07), which corresponds to a haploid nuclear genome size of approximately 820 Mbp. Estimated 2C genome sizes in Apocynaceae range from 0.74 pg in *Voacanga grandifolia *to 5.00 pg in tetraploid *Ceropegia woodii *[59], and correspond to haploid nuclear genome sizes of approximately 360 - 2,445 Mbp. Among the ca. 3,000 species of Asclepiadoideae [60], which include *C. woodii*, other 2C values for diploids are all greater than 4.30 pg [59]. The genome of *Asclepias *is on the small side for the family based on current knowledge, but a comparison is difficult to make due to the limited sampling of genome size diversity in Apocynaceae.

### Illumina sequencing and quality filtering

Of the 10,512,738 reads obtained, a total of 9,651,427 short reads passed the first step of quality filtering yielding 386 Mbp of sequence. The raw reads were submitted to the NCBI Sequence Read Archive (SRP005621). Trimming reads of nucleotides with a "B" quality score and subsequent bases resulted in retention of 99.06% of the quality-filtered data. Reads yielding no data due to all unscorable bases accounted for 0.20% of this read pool. Of the reads obtained, approximately 11.8%, 3.4%, and 1.8% originated from the chloroplast genome, mitochondrial genome, and rDNA repeat arrays respectively. Based on the estimated genome sizes, approximately 0.5× coverage, on average, of the whole *A. syriaca *genome (including organellar genomes) and 0.4× coverage of the nuclear genome were obtained from one lane of Illumina reads.

### Characterization of the organellar genomes of *Asclepias syriaca*

#### The Chloroplast Genome

**Figure 1:**
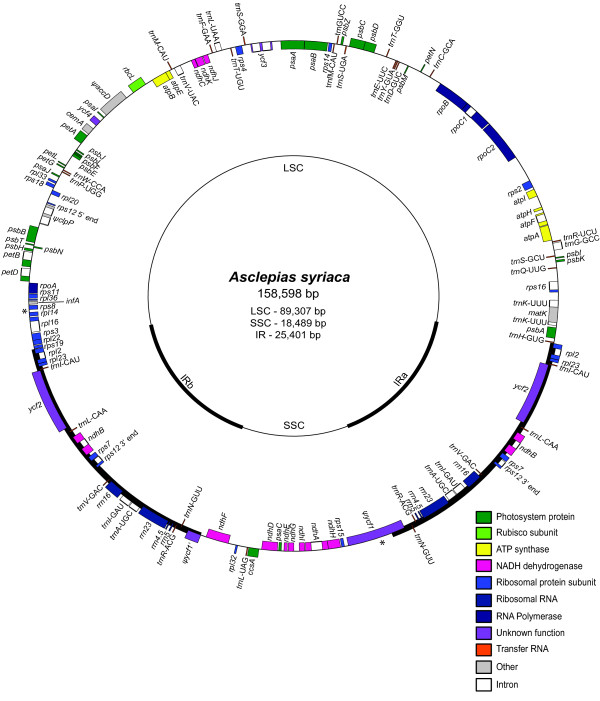
**Map of the chloroplast genome of *Asclepias syriaca***. The thick black lines indicate the locations of the inverted repeats (IR). The thin black lines indicate the locations of the large single copy (LSC) and small single copy (SSC) regions. Transcription is clockwise for genes on the outside of the circle and counterclockwise for genes on the inside of the circle. Asterisks denote the locations of unresolved sequence due to polynucleotide stretches.

The chloroplast genome of *A. syriaca *is 158,598 bp [GenBank:JF433943], excluding two small, unresolved regions that were not able to be assembled or Sanger sequenced due to polynucleotide stretches (*rps8-rpl14 *intergenic spacer and *ψycf1*), and has an inverted repeat (IR) of 25,401 bp (Figure 1). The initial assembly produced using the alignreads pipeline and the oleander reference contained 51 contigs with a median read depth of 246× and N50 of 4,683 bp. The longest contig was 14,884 bp. The final assembly using the finished *A. syriaca *sequence as a reference contained 22 contigs, had an N50 of 9,030 bp, and longest contig of 28,186 bp. The use of chloroplast contigs from 80 bp reads from other *A. syriaca *individuals (S. Straub and A. Liston, unpublished data) in combination with Sanger sequencing of select regions in the 0.5× genome individual and these other individuals, resulted in the addition of 6,622 bp and deletion of 1,402 bp of sequence. That large insertions and deletions were found relative to the original assembly was expected due to the limited power of reference guided assembly algorithms to reconstruct these differences. Only 38 bp of sequence from the original reference guided assembly were determined to be incorrect, producing substitution errors in the genome sequence. The sum total of these changes resulted in the alteration of approximately 5% of the total sequence length, indicating that 95% of the chloroplast genome of *A. syriaca *was assembled at 0.5× average genome coverage with reads of only 40 bp and an oleander reference with 85% sequence identity (considering only one copy of the inverted repeat). Although the original assembly was very good overall, comparison of the final assembly with the finished reference sequence highlighted the limitations of using 40 bp vs. 80 bp reads for sequence assembly. Even with a correct reference sequence, some regions were still not assembled properly (e.g., *accD, ycf1*) and some assembly mistakes from the original assembly were recreated (e.g., an erroneous 5 bp deletion in the middle of *rbcL *causing a frameshift).

In terms of overall size and IR size, *A. syriaca *has a typical asterid chloroplast genome [61]. The gene content of the *A. syriaca *chloroplast genome is similar to those of oleander and coffee (*Coffea arabica*) [62], with 110 unique genes (76 protein coding, 30 tRNA, 4 rRNA). Seventeen of these are fully duplicated in the IR for a total of 127 putatively functional genes. The most notable difference between the milkweed genome and the oleander and coffee chloroplast genomes is that *clpP, accD*, and *ycf1 *are likely pseudogenes in *Asclepias*. Another difference in the *Asclepias *genome relative to the other two genomes is shifting of the boundary of the IR in *A. syriaca *such that the truncated version of *rps19 *observed in oleander and coffee is not present in IRa of milkweed. Assessment of smaller repeats showed that the *A. syriaca *chloroplast genome contains few repeats (Table 5). In comparison, the coffee chloroplast genome has nearly three times as many repeats of 30 or more basepairs with 90% or greater sequence identity [62].

**Table 5:** Repeats in the *Asclepias syriaca *chloroplast genome

Repeat Location(s)	Repeat Type	Repeat Length (bp)	Identity
*trnS*-GCU:*trnS*-GGA	Inverted	32	100%
*ψaccD*	Direct	63	89%
*ψaccD*	Direct	202	85%
*psbN-psbH *spacer	Direct	66	99%
*rpl23-trnI*-CAU spacer	Direct	57	97%
*trnN*-GUU-*ψycf1 *spacer	Direct	299	87%

Repeats of at least 30 bp and at least 85% sequence identity to each copy are reported.

The *A. syriaca ψclpP *has several features that indicate that it is likely a pseudogene. The nucleotide and protein sequences are divergent from those of oleander (81.3% exon nucleotide and 67.0% protein sequence identity) and coffee (80.8% exon nucleotide and 67.5% protein sequence identity). In comparison, oleander and coffee have 97.2% exon nucleotide and 98.9% protein sequence identity. There is also a 28 bp insertion near the end of exon 3 of *A. syriaca *(validated by Sanger sequencing) that causes a frame shift and the introduction of a stop codon. Sequence comparisons showed that *clpP *in oleander and coffee are likely under negative selection (Ka/Ks = 0.07 and 0.03 respectively); however, the Ka/Ks for *Asclepias *is 1.15 indicating that this locus is likely evolving neutrally. While the *clpP *introns have been lost multiple times in angiosperms [e.g., [63-65,67,68]], the coding region has been lost much less often. Other examples of loss of the coding region are only known from *Scaevola *(Goodeniaceae), *Passiflora *(Passifloraceae), *Trachelium *(Campanulaceae), *Geranium *and *Monsonia *(Geraniaceae) [67-69].

The *accD *gene of *A. syriaca *is also likely a pseudogene. This gene is 2,584 bp in *A. syriaca *compared to 1,497 bp in oleander. There is a repeat-rich (Table 5), approximately 1 kb insertion in the middle of the gene. Although the insertion does not interrupt the reading frame, the remainder of the sequence that is alignable with oleander is highly divergent. This gene can be highly variable, even within a genus [66], but due to the extent of sequence divergence, and lengths of the insertion and repeat motifs, this gene is likely non-functional in *Asclepias*. However, in pepper, a smaller, repeat-containing insertion of 144 bp in *accD *does not cause a frameshift nor prevent transcription of the gene [70]. There are numerous other examples of loss of *accD *from the plastome among angiosperms [e.g., [64,65,67-69,71]].

The assembly of *ψaccD *proved especially challenging for *Asclepias *due to sequence divergence and the large insertion relative to the oleander reference. *De novo *assembly of 80 bp reads from another *A. syriaca *individual and Sanger sequencing confirmed the correct sequence. Further inspection indicated that the assembly errors in this region arose due to the presence of an *accD *pseudogene in the mitochondrial genome of *A. syriaca*, also confirmed by Sanger sequencing, which was more similar in sequence and length to the oleander chloroplast *accD*. A similar problem involving *accD *and a mitochondrial genome sequence was encountered in the assembly of the pepper plastome [70]. Problems of this sort are likely to arise for assembly of chloroplast genomes, especially with short reads, for regions of the genome that have been duplicated in the mitochondrial or nuclear genomes [72].

A third possible pseudogene in the *A. syriaca *chloroplast genome is *ψycf1*. This gene contains one of the two polynucleotide repeats of unresolved length in the genome and has a highly divergent sequence compared to oleander and coffee. The sequence of this gene was also checked and improved using *de novo *contigs and Sanger sequencing to eliminate the possibility of assembly errors as a cause of sequence divergence. Although *ycf1 *is one of the most quickly evolving chloroplast genes in seed plants [e.g., [66,73,74]], the full length copy of *ycf1 *in *Asclepias *is so divergent that it may be non-functional. The sequence identity between *ycf1 *in oleander and coffee is 87.5%, whereas the sequence identities between each of those species and *Asclepias *are only 79.4% and 72.2% respectively. In addition to decreased sequence identity, there were 62 and 61 indels inferred from alignments of the *Asclepias ycf1 *sequence with the oleander and coffee sequences respectively, whereas there were only 16 indels inferred from an alignment of oleander and coffee. Among angiosperms *ycf1 *has also been lost multiple times [e.g., [65,67-69,71]].

All three of the putative pseudogenes detected in the *A. syriaca *plastome, *ψclpP, ψaccD*, and *ψycf1 *have functions that are known to be essential for plant development and survival [75-77]. Additional studies to evaluate the functionality of these putative pseudogenes will be needed to confirm their designation as such, in combination with further analysis of the nuclear genome to locate functional copies of these indispensible genes or determine which other nuclear genes might encode a functional replacement as in, for example, the case of *accD *in grasses [e.g., [78,79]].

#### The Mitochondrial Genome

A total of 130,764 bp of the *A. syriaca *mitochondrial genome was assembled in 115 contigs with a median read depth of 32× and N50 of 1,666 bp. See additional file 4: *Asclepias syriaca *mitochondrial genome contigs (also accessible at http://milkweedgenome.org). The longest contig was 6,948 bp. The extent to which the portion of the genome assembled for *A. syriaca *represents the whole mitochondrial genome is unknown due to the extensive variation observed for genome size, gene content, and organization among angiosperms [43,80,81]. Divergence from the organization of the *Nicotiana *reference likely prevented a more complete and contiguous assembly. Complete (*rrn5*) or nearly complete (*rrn26, rrn18*) ribosomal DNA sequences were identified among the contigs. Twenty-eight putative tRNA sequences representing 25 different tRNAs were also identified. Absence of a complete complement of tRNAs was not unexpected because the number of tRNAs in plant mitochondrial genomes is variable due to the replacement of native mitochondrial tRNAs with versions of chloroplast origin or import of nuclear encoded tRNAs of chloroplast or nuclear origin [80,81]. For example, among the tRNAs in the *A. syriaca *mitochondrial genome, both a mitochondrial version and a chloroplast version of *trnE-*TTC were detected.

The protein coding gene content observed for *A. syriaca *was compared to that of the mitochondrial genome of *Nicotiana tabacum *because it is the only other asterid with a sequenced mitochondrial genome. The assembled part of the genome contained the same 37 protein coding genes of known function as the *N. tabacum *mitochondrial genome with an average coverage of 94.8%. Putative pseudogenes of *rps2, rps7*, and *rps14 *were identified in the *A. syriaca *mitochondrial genome contigs. Both the mitochondrial genome of *N. tabacum *and *A. syriaca *appear to lack functional copies of these three genes, in addition to lacking *rpl6, rps8*, and *rps11*, the genes lost in all angiosperms, but present in *Marchantia *[42]. Evidence of *ψrps14 *is present in tobacco, but there is no remaining evidence of the other two genes. Due to the pseudogenization or absence of these genes in the *N. tabacum *reference used for the *A. syriaca *assembly, a more complete characterization of the mitochondrial genome based on additional data will be required to determine if these genes are truly non-functional in *A. syriaca *or if their absence is an assembly artifact.

Extensive horizontal gene transfer has been hypothesized for the group I intron present in the *cox1 *gene of angiosperm mitochondrial genomes, with six horizontal transfer events hypothesized in Apocynaceae alone [82]. Alternatively, it has been argued that the distribution of the *cox1 *intron in plants more likely involved a single horizontal transfer followed by intron loss in many lineages [83]. All but one of the Apocynaceae genera sampled have contained the *cox1 *intron [82,83], and it was also found to be present in the assembled *cox1 *gene of *A. syriaca*. This result adds to the evidence supporting the intron loss hypothesis over horizontal gene transfer in Apocynaceae.

### Characterization of the nuclear genome of *Asclepias syriaca*

The Velvet *de novo *assembly using a hash length of 21 resulted in 1,399,320 bp of sequence in 6,997 contigs, 79% of which were 200 bp or less. See additional file 5: *Asclepias syriaca *nuclear genome *de novo *assembly contigs (also accessible at http://milkweedgenome.org). The longest contig was 5,027 bp and the N50 for the assembly was 196.

#### Repetitive Element Characterization

**Figure 2:**
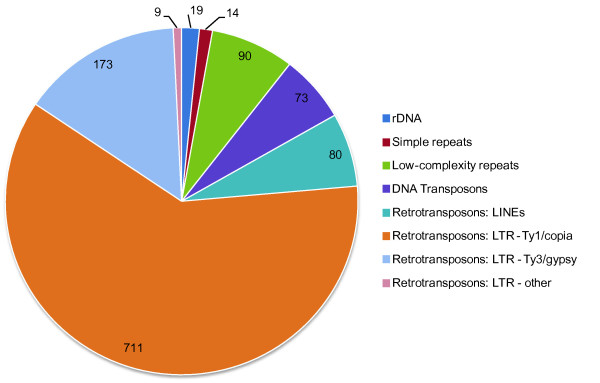
**Repeat types detected among *de novo *contigs of the *Asclepias syriaca *nuclear genome**. The numbers indicate individual hits in the contig sequences.

A total of 564 repetitive elements were detected in the *A. syriaca de novo *contigs (Figure 2). The majority of these repeats were Ty1/*copia-*like and Ty3/*gypsy*-like LTR retrotransposons (Figure 2), which are abundant in plants and common across eukaryotes [84-86]. Only the most highly represented repeats in the genome would have high enough coverage at 0.4× total nuclear genome coverage to be assembled, so it is not surprising that the majority of the repeats were Ty1/*copia-*like and Ty3/*gypsy*-like retrotransposons. In plant species where retrotransposons have been characterized, it appears to be lineage specific whether Ty1/*copia*-like or Ty3/*gypsy*-like retroelements are more abundant in the genome, likely because retrotransposons can proliferate or be lost from genomes over relatively short evolutionary time scales [85,87]. Among other asterids, Ty3/*gypsy*-like retrotransposons are more prevalent in the sunflower (*Helianthus annuus*), tomato, and potato genomes, while in carrot (*Daucus carota*), as in *A. syriaca*, Ty1/*copia*-like retrotransposons are more numerous [88-90].

The numbers of retroelements reported here are certain to be different than the actual numbers of retroelements in the sampled *A. syriaca *genome because the *de novo *contigs were relatively short compared to the length of retrotransposons, possibly resulting in different regions of the same retroelement being counted multiple times. Additionally, six contigs had more than one good repeat type match, slightly inflating the numbers discovered. Only approximately 2% of the putatively nuclear reads had hits to the repeat-containing contigs. The estimate of raw reads with hits to the plant repeat database did not give a better estimate of the total repeat content of the *A. syriaca *genome than the hits to assembled repeats with only 0.24% of reads with one or more hits. These results indicate that at 0.4× genome coverage, only an initial characterization of the most common repeats present in a plant genome can be made, while an estimation of the overall repeat content of the genome requires deeper coverage.

Microsatellite repeats were also detected among the 2,624 *de novo *contigs of at least 150 bp, with a total of 395 microsatellite sequences found in 333 of these contigs (Table 6). Top ten BLAST hits with an E-value of 1e^-5 ^or lower were recovered for 53 and three contigs for mitochondrial and chloroplast sequences respectively. The higher number of possible mitochondrial reads remaining in the read pool following removal of reads putatively belonging to the organellar genomes reflects our more limited knowledge of the mitochondrial genome in plants in general and incomplete assembly of the *A. syriaca *mitochondrial genome. An alternative explanation for the presence of these small segments of DNA of mitochondrial or chloroplast origin in putatively nuclear contigs is that they correspond to nuclear nomads [72].

**Table 6:** Microsatellite loci detected in the 0.5× coverage genome of *Asclepias syriaca*

Repeat Type	Number Found	Number of Loci with Primers	Number Tested/Successful for PCR Amplification
di-	271	206	17/17
tri-	51	30	7/7
tet-	57	43	1/1
pent-	12	9	0/NA
hex-	4	2	0/NA

**Total**	**395**	**290**	**25/25**

Amplification success of the 25 markers with the highest numbers of repeat units is indicated (see Table 2).

To be conservative in selecting 25 candidates for testing, loci from the 56 contigs with putatively mitochondrial or chloroplast sequence were removed from the pool of candidates, leaving 209 primer sets for putatively nuclear microsatellite loci. See additional file 2: Primer sets designed using BatchPrimer3 for 184 nuclear microsatellite loci in *Asclepias syriaca *not tested for amplification success. Of the 25 primer sets tested, all successfully amplified in *A. syriaca *(Table 6). It is important to note that with the low overall genome coverage, at least some of these microsatellite-containing contigs are likely to have originated from multiple, nearly-identical paralogous loci. As is the case with all microsatellites, the ultimate proof in the utility of these markers for identifying intraspecific variation (e.g., linkage mapping or population genetics) will come from obeying a Mendelian locus/allele model that meets expected transmission ratios (in the case of known pedigrees) or Hardy-Weinberg expectations (in the case of wild populations). If the markers show evidence of fixed heterozygosity or dominant-only variation, this would constitute evidence that our microsatellite 'contigs' are the product of microreads representing two or more paralogs. These loci are currently being examined in greater detail to determine their locus specificity.

The source of the rDNA repeats identified by RepeatMasker in the *de novo *contigs (Figure 2) that remained after the removal of *A. syriaca *rDNA microreads was also explored. BLAST searches (blastn) of the NCBI nucleotide collection revealed that their source was likely plant fungal pathogens or endophytes. For example, the top BLAST hits for two of theses contigs were to an *Alternaria alternata *gene for large subunit (LSU) rRNA (1e^-105^) [GenBank:AB566324.1] and a *Davidiella tassiana *partial LSU rRNA gene sequence (1e^-92^) [GenBank:FN868880.1]. Approximately 0.02% of the total reads could be categorized as fungal rDNA when the sequences for the top GenBank hits were used as a BLAT database. Detection of fungal contaminants of a plant genomic DNA preparation is unsurprising due to the ubiquitous association of these two types of organisms.

#### Ribosomal DNA

**Figure 3:**
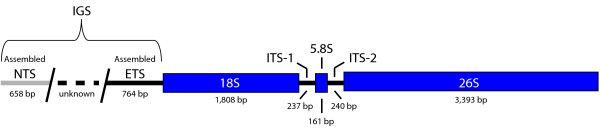
**The rDNA cistron of *Asclepias syriaca***. Blue boxes represent genes. Thick black lines represent additional transcribed sequence. The gray and dashed lines represent non-transcribed and unassembled sequence respectively. Only partial sequences of the non-transcribed spacer (NTS) and external transcribed spacer (ETS) were able to be assembled due to repeats, so the length of the intergenic spacer (IGS) remains unknown.

A 385 bp contig containing a 120 bp 5S rDNA sequence [GenBank:JF312047] and an rDNA cistron of 7,541 bp [GenBank:JF312046] were assembled for *A. syriaca *(Figure 3). Repeat structure in the external transcribed spacer (ETS) and intergenic spacer (IGS) regions of the rDNA cistron prevented further extension of that contig and produced an error in the assembly where reads corresponding to similar repeats were incorrectly piled up, highlighting one of the primary difficulties of genome assembly using short reads [32,91]. Consequently, the first 280 bp of the contig sequence were removed prior to downstream analyses. The median read depth for the final assemblies were 406× for 5S and 738× for the 18S-5.8S-26S cistron. Using the median read depth as an estimate of the number of rDNA copies sequenced and the nuclear genome coverage of approximately 0.4×, rough approximations of the number of 5S rDNA repeats and of rDNA cistron copies are 1,015 and 1,845 respectively. These estimates are in line with values observed for other plants [92,93].

Fully characterized rDNA cistrons in plants have at least one TATA box upstream of 18S, which demarcates the boundary between the ETS region and the repeat-rich non-transcribed spacer [94,95]. However, repeat units may also appear between the transcription initiation site (TIS) and 18S [96]. The conserved plant TIS sequence was not observed in the *A. syriaca *sequence upstream of 18S, so the entire ETS region was not assembled due to internal repeated sequence. However, another ETS motif conserved in plants and always located approximately 420 - 450 bp upstream of the start of 18S [TGAGTGGTGG; [97]] was located 423 bp upstream of 18S in the sequenced portion of ETS. Due to the length variation in the ETS regions in other asterids (*Solanum *~1000 bp; *Nicotiana *>3000 bp in *N. tabacum*; *Olea *~2000 bp), it is difficult to estimate the completeness of our assembly for this region and the cistron as a whole, which is between 9 and 12 kb in *Nicotiana *[96,98,99].

**Figure 4:**
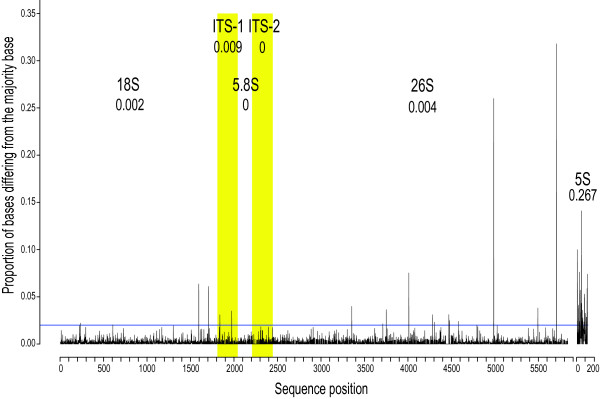
**Polymorphism among rDNA cistron (18S-5.8S-26S) and 5S ribosomal DNA copies in *Asclepias syriaca***. Non-zero proportions below the blue line are likely due to sequencing error. Proportions above the blue line likely represent polymorphism within or among rDNA arrays. The proportion of polymorphic sites for each rDNA region is given beneath the label.

For intragenomic rDNA polymorphism characterization, quality filtering by average quality (q = 20) removed zero reads from the 5S pool, and 148 reads from the 18S-5.8S-26S cistron pool (0.1%). Masking the remaining nucleotides with qualities below 20 affected 1.7% and 6.2% of nucleotides in the two pools, respectively. A high percentage of positions in the 5S locus were polymorphic (Figure 4). Most of these positions had less than 8% of reads differing from the consensus, though some ranged as high as 14%. Conversely, only 19 positions in the 18S-5.8S-26S cistron were polymorphic (Figure 4). Polymorphisms were found in both coding and spacer regions, although none were found in either the 5.8S or ITS2 regions. While spacer regions contained a slightly higher proportion of polymorphic positions than coding regions, positions with the greatest proportion of differing reads were found within coding regions, in particular in the 26S region.

Levels of intra-genomic polymorphism among copies of the rDNA loci generally agree with previously measured and theoretically expected levels. The high proportion of polymorphic positions in 5S is concordant with previous observations for this locus; however, the levels measured here are somewhat higher than those reported in other plant and animal species [e.g., [100-102]]. Intra-genomic polymorphism among copies of the rDNA cistron in this individual is comparable to rates found in previous studies characterizing polymorphism within *Drosophila *[103] and fungi [104]. While the 19 sites found in this study are within the previously observed range of variation, comparisons are difficult due to the difference in copy number between these taxa (45-180 in the fungi, 200-250 in *Drosophila*, approximately 1800 in this study), differences in the definition of polymorphic loci, and differences in sequencing technologies (whole-genome shotgun sequencing vs. Illumina).

The distribution of polymorphic positions matches theoretical expectations, with a higher proportion of positions found within spacer rather than coding regions. The very high proportion of differing reads found at two positions in the 26S region may be caused by their location within expansion segments of this region, expansion segments 9 and 8, respectively [105]. The lower levels of polymorphism observed for the 18S-5.8S-26S cistron relative to the 5S rDNA loci are concordant with the molecular evolutionary processes associated with each rDNA type. While strong concerted evolution and strong purifying selection act to homogenize the 18S-5.8S-26S cistron copies within and among repeat arrays, weak concerted evolution and birth-and-death processes for 5S rDNA allow accumulation of polymorphism [100,106].

#### Protein Coding Nuclear Genes

**Figure 5:**
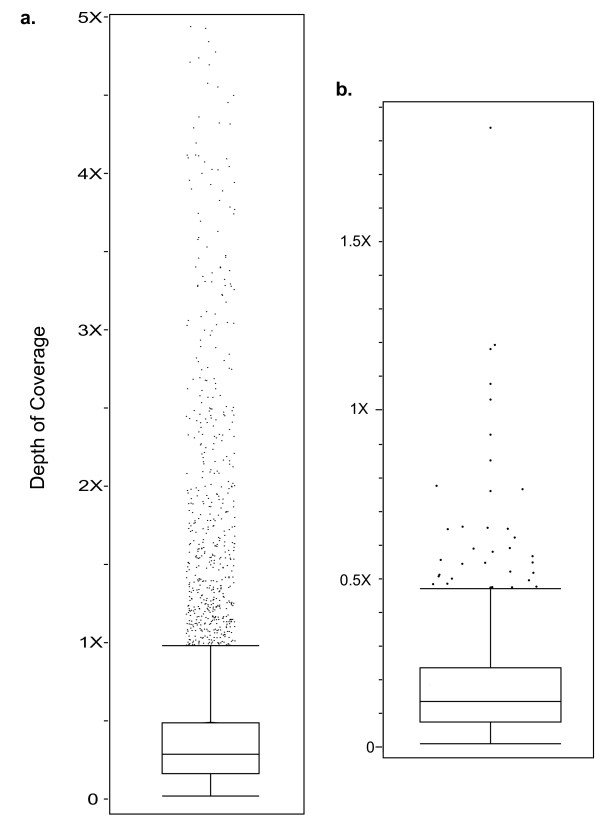
**Coverage of *Catharanthus* unigenes and COSII markers by *Asclepias syriaca* microreads**. a. Coverage of *Catharanthus* nuclear unigenes by *A. syriaca* microreads for genes with less than 5X coverage. Fifty-six of the 8,077 unigenes with coverage greater than or equal to 5X were excluded from the figure. Among these, the maximum coverage was 17.5X.   b. Coverage of COSII single-copy nuclear genes by *A. syriaca* microreads.  The upper box plot whisker represents 1.5 times the interquartile range and the lower whisker extends to the minimum coverage value. Dots represent outliers.

From 19,899 *Catharanthus *ESTs, 9,266 unigenes were created. Of these, 72 corresponded to chloroplast, mitochondrial or rDNA genes and were removed from the data set. Among the remaining repeat masked unigenes, 8,077 had between one and 138 unique microread hits. The median number of hits per kilobase of sequence was 7.17 (95% CI 7.03 - 7.29). The median coverage of the unigenes was 0.29× (95% CI 0.28× - 0.29×; Figure 5a). The 618 genes with coverage levels above 0.984× were outliers. Many of these genes likely correspond to members of multigene families or other duplicated genes, increasing the chances of non-orthologous BLAT hits in conserved domains. For example, a unigene with 6.79× coverage had multiple BLAST hits (E = 0) to eudicot H^+^-ATPases (P_3A_-type). Within the P-type ATPase gene super family, the P_3A_-type subfamily has 10 and 11 members in rice and *Arabidopsis *respectively [107]. Based on the overall genome coverage and the unigene coverage, the *A. syriaca *genome likely contains a similar number of P_3A_-type ATPases.

Another explanation for the high coverage of outlier loci was indicated by the masked unigene sequences themselves. Many of the unigenes with the highest coverage still contained sequences that could produce multiple non-homologous hits (e.g., internal polynucleotide stretches), which in turn produced stacks of reads. Read overlap due to this phenomenon inflated the coverage values, so many of the very high coverage values are likely artifacts of the unigene creation and masking process. The presence of PCR duplicate reads in the data set could also have caused read overlap and overestimation of coverage. Most of these reads should have been excluded by counting only unique BLAT hits, but any sequencing errors in the duplicates or length differences due to differing sequence quality, especially at the ends of the reads, could produce nearly identical hits.

Of the 1,086 COSII alignments 721 had between one and 23 unique *A. syriaca *microread hits with a median gene coverage of 0.14× (95% CI 0.13× - 0.15×; Figure 5b) and median hits per kilobase of sequence of 3.40 (95% CI 3.13 - 3.64). The 35 genes with greater than 0.473× coverage were outliers. There is a possibility that some of these genes are single copy in other asterids, but duplicated in *Asclepias*, leading to the higher coverage levels. More likely, as in the unigene analysis, sequence features, including microsatellites that were not masked in the COSII alignments, and PCR duplicates led to increased estimates of coverage. Of the five genes with greater than 1× coverage, the only BLAT hits for two were to microsatellites and a third had multiple microsatellite and a single other hit.

The median coverage values for the unigene and COSII data sets would be expected to be close to the estimate of 0.4× coverage for the nuclear genome. However, the observed values deviated from the expected coverage. One explanation for this is that the overall coverage of the *A. syriaca *genome was slightly overestimated due to the presence of PCR duplicates in the read pool. It is also possible that the genome size of the sequenced individual was larger than the genome sizes estimated from the experimental population, again causing an overestimate of genome coverage. Divergence from the reference sequences would also cause an underestimate of coverage for *A. syriaca *orthologs. The lower amount of divergence from *Catharanthus*, a member of the same family, is reflected in the higher estimated coverage for the unigenes than the COSII alignments, which contained sequences of more distantly related asterids. The coverage value for the unigenes may also be higher due to the reasons discussed above. Another feature of both reference data sets that would cause an underestimate of coverage was the absence of introns. The microread sequences from genomic DNA would also represent these features of the genes, but would not produce BLAT hits for these genes. Reads spanning intron-exon boundaries would also have been excluded in many cases due to the mismatch of the intron sequence with the following exon in the unigenes and COSII alignments. Even with these issues confounding coverage estimates, these two explorations of the nuclear gene content of the *A. syriaca *genome demonstrate that even at 0.4× genome coverage, much of the protein coding portion of the nuclear genome can be detected.

Even with low overall coverage of the 721 COSII genes with *A. syriaca *microread hits, the alignments were still useful for marker development for *Asclepias *and Apocynaceae molecular phylogenetics. The 28 genes that had 0.5× or greater coverage were evaluated for their suitability for this purpose. Microreads flanked putative intron locations in 15 genes of this set, allowing primer design for amplification of one or more introns per gene (Table 3).

Primer testing within *Asclepias *and across Apocynaceae was largely successful with single products amplifying in most tribes of Apocynaceae (Table 7). Amplification of large introns and evidence of length variation among groups are promising for utility of these markers at different scales dependent upon overall sequence divergence. These results highlight how even a small amount of genomic data can facilitate and expedite the arduous process of nuclear marker development across a range of taxonomic scales from within a genus to across a family for groups of species not closely related to a species with extensive genomic resources.

**Table 7:** Success of PCR amplification and intron length variation for COSII nuclear loci in *Asclepias *and across Apocynaceae

					Number of Individuals PCR successful/PCR attempted					Approximate Product Size (bp)#				
					
*Arabidopsis *Ortholog	tair Annotation	Primer Set	Introns spanned*	Coding Sequence (bp)	As^1^	M^2^	B^3^	Ap^4^	Al^5^	As	M	B	Ap	Al
At1g24360	3-oxoacyl-(acyl-carrier protein) reductase, chloroplast	At1g24360a	360, 441, 487	234	15/20	1/2	1/2	2/2	1/1	1400-1500	1500	1000	300, 1000	1000
		At1g24360b	582, 615	117	14/16	2/2	2/2	2/2	1/1	500-700	500	500	500	700, 1500
		At1g24360c	750	117	16/16	2/2	2/2	2/2	1/1	200	200	200, 1500	200	200, 700
														

At1g44760	universal stress protein (USP) family protein	At1g44760a	369, 491	334	1/16	1/2	1/2	2/2	1/1	1300	1200	1200	1100	700

At1g55880	pyridoxal-5'-phosphate-dependent enzyme	At1g55880a	1043	394	13/16	2/2	2/2	2/2	1/1	1200	900	700 - 800	700	300, 700

At1g77370	glutaredoxin, putative	At1g77370a	164, 238	172	16/20	2/2	2/2	0/2	0/1	1400-1500	1100	1100 -1200	n/a	n/a

At2g03120	homologous to Signal Peptide Peptidases (SPP)	At2g03120a	156	210	19/19	2/2	2/2	2/2	1/1	300-400	300	300	300	300

At2g06530	VPS2.1 (vesicle-mediated transport)	At2g06530a	276	145	20/20	2/2	2/2	2/2	1/1	400-650	400	700 - (600, 1000)	600 - (800,1000)	400, 1200

At2g21960	unknown protein	At2g21960a	503, 592, 722	366	7/16	2/2	2/2	2/2	1/1	1500	1500	1700	1600 - 1700	1100
		At2g21960b	813	84	12/16	2/2	2/2	2/2	1/1	200-300	200	150	150	150

At2g30200	[acyl-carrier-protein] S-malonyltransferase	At2g30200a	377, 444	185	12/16	2/2	2/2	2/2	1/1	1400	1000	1200	900	1200, >>1500
		At2g30200b	587, 636, 774	392	13/20	2/2	2/2	2/2	1/1	700-1500	1200	1200	1200	1200
		At2g30200c	855	160	16/16	0/2	2/2	2/2	1/1	300	n/a	300	300	300

At2g34620	mitochondrial transcription termination factor-related	At2g34620a	596	254	19/19	2/2	2/2	2/2	2/2	300	300	300	300 - 1000	300
		At2g34620F/	596	531	15/16	2/2	2/2	2/2	0/2	600	600	600	600	n/a
		At2g34620aR												

At2g47390	serine-type endopeptidase	At2g47390a	1718	401	20/20	2/2	2/2	2/2	0/2	500-600	500	500	500	n/a
		At2g47390b	2177, 2243, 2304, 2454, 2592	506	1/16	0/2	0/2	0/2	0/2	1500	n/a	n/a	n/a	n/a

At4g13430	methylthioalkylmalate isomerase	At4g13430a	510, 579, 696	275	14/16	2/2	2/2	2/2	1/1	700-1000	900, 1200	1200	1200	1200
		At4g13430b	771, 849, 945, 995, 1050	391	16/20	0/2	2/2	0/2	1/1	1300-1500	n/a	>1500	n/a	1500
		At4g13430c	1182, 1230	278	15/16	2/2	2/2	2/2	1/1	(500, 800)-1000	800	900 - >1500	800	500, 900, 1200

At4g26750	hydroxyproline-rich glycoprotein family protein	At4g26750a	305, 381	241	1/16	2/2	2/2	2/2	0/1	1500	>1500	1500	1000	n/a

At5g13420	putative transaldolase	At5g13420a	348, 398	295	12/16	2/2	2/2	2/2	0/1	1300	1100	1000	700	n/a
		At5g13420b	699, 888	338	20/20	2/2	2/2	2/2	1/1	800-900	1200	1200	500, 1200	1500

At5g23540	26S proteasome regulatory subunit	At5g23540a	308, 390, 567	436	0/16	2/2	2/2	1/2	0/1	n/a	>>1500	300, 700, >>1500	500, 700	n/a
		At5g23540aF At5g23540bR	308, 390	231	7/16	2/2	2/2	1/2	1/1	1400	>>1500	700 - 900, >>1500	700	1200

At5g49970	bifunctional pyridoxine (pyridoxamine) 5'-phosphate oxidase (PPOX)	At5g49970a	507	193	20/20	2/2	2/2	2/2	2/2	300	300	300	300	300
		At5g49970b	613, 780	226	16/16	2/2	2/2	2/2	1/2	500-900	800	800	700	1000
		At5g49970aFAt5g49970bR	507, 613, 780	398	15/16	n/a	n/a	n/a	n/a	1000-1500	n/a	n/a	n/a	n/a

*Intron numbers represent the starting position of the intron in *Arabidopsis*.

^1^Asclepiadinae Samples include 4 genera: *Asclepias *(17), *Gomphocarpus, Calotropis*, and *Pergularia*.

^2^Marsdenieae

^3^Baisseeae

^4^Apocyneae

^5^Alyxieae

#Two numbers separated by a comma indicate multiple PCR products. A range of numbers indicates size variation among individuals.

## Conclusions

The often-employed strategy of transcriptome sequencing for exploration of the genomes of non-model organisms can produce a wealth of information for comparative and functional genomics studies due to high coverage of the gene space and provide the necessary information for marker development, including single nucleotide polymorphisms [3]. As an alternative, low coverage whole genome sequencing provides a more complete survey of the entire genome by providing substantial information about the organellar genomes and the repeat content of the nuclear genome, while still providing the necessary resources for marker development. In plants with no prior genomic information, acquisition of even a small amount of genomic data allows ready characterization of the high copy fraction of the genome and exploration of the low copy fraction of the genome. A complete, or nearly complete, chloroplast genome and rDNA cistron are readily obtained. A partial to nearly complete mitochondrial genome can be obtained, depending on the complexity of the particular mitochondrial genome. Initial characterizations of the repeat and protein coding portions of the nuclear genome can be made in terms of content and then used in the development of molecular tools, including low-copy nuclear genes and microsatellites, for the further study of the target organism and its relatives.

The data obtained through this study are the first step in the development of a community resource for further study of plant-herbivore and plant-pollinator interactions, floral developmental genetics, chemical evolution, population genetics, and comparative genomics using milkweeds as ecological and evolutionary models. The information obtained from this study is already serving as a resource for development of markers to aid in the study of phylogenetics, population genetics, phylogeography and other areas for *Asclepias *and Apocynaceae. These results highlight the promise of genomic resources for developing tools in any organism [2,108] and show that even labs with limited resources can feasibly obtain 0.5× or greater coverage of the genome of their species of interest and a wealth of information. By incorporating 80-120 bp read lengths, paired-end sequences, and ever-improving sequencing technology, this level of coverage and assembly success from a single lane of Illumina sequencing is attainable for all but the very largest of plant genomes.

## Authors' contributions

SCKS participated in study design, conducted the majority of the analyses, designed the primers, collected the chloroplast Sanger sequence data and drafted the manuscript. MF conceived of the study and conducted microsatellite and low-copy nuclear gene primer testing in *Asclepias*. TL participated in the design of the low-copy nuclear gene analyses and conducted low-copy nuclear gene primer testing in Apocynaceae. ZF scripted and tested the data analysis pipeline. MP prepared the Illumina library and tested the data analysis pipeline. KW conducted the rDNA polymorphism analyses. RCC conceived of the study, performed some statistical analyses, and collected the genome size data. AL conceived of the study, coordinated the data collection and study design, conducted the COSII nuclear gene analysis and assisted in some of the other analyses. All authors read and approved the final manuscript.

## Supplementary Material

Additional file 1 **Polymorphism detected among Illumina reads for the PhiX sequencing control**. A graph showing the proportion of bases differing from the majority base in the Illumina PhiX sequencing control. Click here for file

Additional file 2 **Primer sets designed using BatchPrimer3 for 184 nuclear microsatellite loci in *Asclepias syriaca *not tested for amplification success**. A table of nuclear microsatellite locus primer sets, including predicted product size and repeat motif information. Click here for file

Additional file 3 **List of GI numbers for 19,899 *Catharanthus roseus *ESTs downloaded from GenBank and assembled into unigenes**. List of GenBank numbers for *Catharanthus roseus *ESTs used in this study. Click here for file

Additional file 4 ***Asclepias syriaca *mitochondrial genome contigs**. FASTA file containing sequences of the assembled *Asclepias syriaca *mitochondrial genome contigs. Click here for file

Additional file 5 ***Asclepias syriaca *nuclear genome *de novo *assembly contigs**. FASTA file containing sequences of the nuclear genome contigs from the Velvet *de novo Asclepias syriaca *genome assembly. Click here for file

## References

[B1] Rokas A Abbot P Harnessing genomics for evolutionary insights Trends Ecol Evol 2009 24 4 192 200 10.1016/j.tree.2008.11.004 19201503

[B2] Hudson ME Sequencing breakthroughs for genomic ecology and evolutionary biology Mol Ecol Resour 2008 8 1 3 17 10.1111/j.1471-8286.2007.02019.x 21585713

[B3] Ekblom R Galindo J Applications of next generation sequencing in molecular ecology of non-model organisms Heredity in press 10.1038/hdy.2010.152 PMC3186121 21139633

[B4] Rasmussen DA Noor MAF What can you do with 0.1× genome coverage? A case study based on a genome survey of the scuttle fly *Megaselia scalaris *(Phoridae) BMC Genomics 2009 10 382 10.1186/1471-2164-10-382 19689807 PMC2735751

[B5] Lee RM Thimmapuram J Thinglum KA Gong G Hernandez AG Wright CL Kim RW Mikel MA Tranel PJ Sampling the Waterhemp (*Amaranthus tuberculatus*) genome using pyrosequencing technology Weed Sci 2009 57 5 463 469 10.1614/WS-09-021.1

[B6] Swaminathan K Alabady MS Varala K De Paoli E Ho I Rokhsar DS Arumuganathan AK Ming R Green PJ Meyers BC Genomic and small RNA sequencing of *Miscanthus *× *giganteus *shows the utility of sorghum as a reference genome sequence for Andropogoneae grasses Genome Biol 2010 11 2 R12 10.1186/gb-2010-11-2-r12 20128909 PMC2872872

[B7] Hribova E Neumann P Matsumoto T Roux N Macas J Dolezel J Repetitive part of the banana (*Musa acuminata*) genome investigated by low-depth 454 sequencing BMC Plant Biol 2010 10 204 10.1186/1471-2229-10-204 20846365 PMC2956553

[B8] Macas J Neumann P Navratilova A Repetitive DNA in the pea (*Pisum sativum *L.) genome: comprehensive characterization using 454 sequencing and comparison to soybean and *Medicago truncatula* BMC Genomics 2007 8 427 10.1186/1471-2164-8-427 18031571 PMC2206039

[B9] Webb KM Rosenthal BM Next-generation sequencing of the *Trichinella murrelli *mitochondrial genome allows comprehensive comparison of its divergence from the principal agent of human trichinellosis, *Trichinella spiralis* Infect Genet Evol 2011 11 1 116 123 10.1016/j.meegid.2010.10.001 20946970

[B10] Willerslev E Gilbert MT Binladen J Ho S Campos P Ratan A Tomsho L da Fonseca R Sher A Kuznetsova T Analysis of complete mitochondrial genomes from extinct and extant rhinoceroses reveals lack of phylogenetic resolution BMC Evol Biol 2009 9 1 95. 10.1186/1471-2148-9-95 19432984 PMC2694787

[B11] Dempewolf H Kane NC Ostevik KL Geleta M Barker MS Lai Z Stewart ML Bekele E Engels JMM Cronk QCB Establishing genomic tools and resources for *Guizotia abyssinica *(L.f.) Cass.--the development of a library of expressed sequence tags, microsatellite loci, and the sequencing of its chloroplast genome Mol Ecol Resour 2010 10 6 1048 1058 10.1111/j.1755-0998.2010.02859.x 21565115

[B12] Meyers S Liston A Characterizing the genome of wild relatives of *Limnanthes alba *(Meadowfoam) using massively parallel sequencing Acta Horticulturae 2010 859 309 314

[B13] Nock CJ Waters DL Edwards MA Bowen SG Rice N Cordeiro GM Henry RJ Chloroplast genome sequences from total DNA for plant identification Plant Biotechnol J 2011 9 328 333 10.1111/j.1467-7652.2010.00558.x 20796245

[B14] Givnish TJ Ames M McNeal JR McKain MR Steele PR dePamphilis CW Graham SW Pires JC Stevenson DW Zomlefer WB Assembling the tree of the monocotyledons: plastome sequence phylogeny and evolution of Poales 1 Ann Mo Bot Gard 2010 97 4 584 616 10.3417/2010023

[B15] Castoe TA Poole AW Gu WJ de Koning APJ Daza JM Smith EN Pollock DD Rapid identification of thousands of copperhead snake (*Agkistrodon contortrix*) microsatellite loci from modest amounts of 454 shotgun genome sequence Mol Ecol Resour 2010 10 2 341 347 10.1111/j.1755-0998.2009.02750.x 21565030 PMC5172459

[B16] Abdelkrim J Robertson BC Stanton JAL Gemmell NJ Fast, cost-effective development of species-specific microsatellite markers by genomic sequencing Biotechniques 2009 46 3 185 192 10.2144/000113084 19317661

[B17] Moore MJ Soltis PS Bell CD Burleigh JG Soltis DE Phylogenetic analysis of 83 plastid genes further resolves the early diversification of eudicots Proc Natl Acad Sci USA 2010 107 10 4623 4628 10.1073/pnas.0907801107 20176954 PMC2842043

[B18] Bai X Zhang W Orantes L Jun T-H Mittapalli O Mian MAR Michel AP Combining next-generation sequencing strategies for rapid molecular resource development from an invasive aphid species, *Aphis glycines* PLoS ONE 2010 5 6 e11370. 20614011 10.1371/journal.pone.0011370 PMC2894077

[B19] Rasmann S Agrawal AA Cook SC Erwin AC Cardenolides, induced responses, and interactions between above- and belowground herbivores of milkweed (*Asclepias *spp.) Ecology 2009 90 9 2393 2404 10.1890/08-1895.1 19769118

[B20] Broyles SB Hybrid bridges to gene flow: A case study in milkweeds (*Asclepias*) Evolution 2002 56 10 1943 1953 12449481 10.1111/j.0014-3820.2002.tb00120.x

[B21] Wyatt R Broyles SB Ecology and evolution of reproduction in milkweeds Annu Rev Ecol Syst 1994 25 423 441 10.1146/annurev.es.25.110194.002231

[B22] Fishbein M Venable DL Evolution of inflorescence design: Theory and data Evolution 1996 50 6 2165 2177 10.2307/2410688 28565680

[B23] Agrawal AA Fishbein M Phylogenetic escalation and decline of plant defense strategies Proc Natl Acad Sci USA 2008 105 29 10057 10060 10.1073/pnas.0802368105 18645183 PMC2481309

[B24] Agrawal AA Fishbein M Plant defense syndromes Ecology 2006 87 7 S132 S149 10.1890/0012-9658(2006)87[132:PDS]2.0.CO;2 16922309

[B25] Malcolm SB Rosenthal GA, Berenbaum MR Cardenolide-mediated interactions between plants and herbivores Herbivores: their interactions with secondary plant metabolites Volume I: The chemical participants 1991 2 San Diego: Academic Press 251 296

[B26] Murata J Bienzle D Brandle JE Sensen CW De Luca V Expressed sequence tags from Madagascar periwinkle (*Catharanthus roseus*) FEBS Lett 2006 580 18 4501 4507 10.1016/j.febslet.2006.07.020 16870181

[B27] Murata J Roepke J Gordon H De Luca V The leaf epidermome of *Catharanthus roseus *reveals its biochemical specialization Plant Cell 2008 20 3 524 542 10.1105/tpc.107.056630 18326827 PMC2329939

[B28] Shukla AK Shasany AK Gupta MM Khanuja SPS Transcriptome analysis in *Catharanthus roseus *leaves and roots for comparative terpenoid indole alkaloid profiles J Exp Bot 2006 57 14 3921 3932 10.1093/jxb/erl146 17050644

[B29] Abzhanov A Extavour CG Groover A Hodges SA Hoekstra HE Kramer EM Monteiro A Are we there yet? Tracking the development of new model systems Trends in Genet 2008 24 7 353 360 10.1016/j.tig.2008.04.002 18514356

[B30] Dolezel J Bartos J Voglmayr H Greilhuber J Nuclear DNA content and genome size of trout and human Cytom Part A 2003 51A 2 127 128 10.1002/cyto.a.10013 12541287

[B31] Solexa, Inc Protocol for Whole Genome Sequencing using Solexa Technology BioTechniques Protocol Guide 2007 2006 New York: BioTechniques 291

[B32] Ratan A Assembly algorithms for next-generation sequence data PhD thesis 2009 The Pennsylvania State University, Computer Science and Engineering

[B33] Delcher AL Phillippy A Carlton J Salzberg SL Fast algorithms for large-scale genome alignment and comparison Nucleic Acids Res 2002 30 11 2478 2483 10.1093/nar/30.11.2478 12034836 PMC117189

[B34] Kurtz S Phillippy A Delcher AL Smoot M Shumway M Antonescu C Salzberg SL Versatile and open software for comparing large genomes Genome Biol 2004 5 2 R12. 14759262 10.1186/gb-2004-5-2-r12 PMC395750

[B35] Ovcharenko I Loots GG Giardine BM Hou MM Ma J Hardison RC Stubbs L Miller W Mulan: Multiple-sequence local alignment and visualization for studying function and evolution Genome Res 2005 15 1 184 194 10.1101/gr.3007205 15590941 PMC540288

[B36] Katoh K Kuma K Toh H Miyata T MAFFT version 5: improvement in accuracy of multiple sequence alignment Nucleic Acids Res 2005 33 2 511 518 10.1093/nar/gki198 15661851 PMC548345

[B37] Wyman SK Jansen RK Boore JL Automatic annotation of organellar genomes with DOGMA Bioinformatics 2004 20 17 3252 3255 10.1093/bioinformatics/bth352 15180927

[B38] Milne I Bayer M Cardle L Shaw P Stephen G Wright F Marshall D Tablet--next generation sequence assembly visualization Bioinformatics 2010 26 3 401 402 10.1093/bioinformatics/btp666 19965881 PMC2815658

[B39] Zerbino D Birney E Velvet: algorithms for *de novo *short read assembly using de Bruijn graphs Genome Res 2008 18 821 829 10.1101/gr.074492.107 18349386 PMC2336801

[B40] Hall TA BioEdit: a user-friendly biological sequence alignment editor and analysis program for Windows 95/98/NT Nucl Acids Symp Ser 1999 41 95 98

[B41] Conant GC Wolfe KH GenomeVx: simple web-based creation of editable circular chromosome maps Bioinformatics 2008 24 6 861 862 10.1093/bioinformatics/btm598 18227121

[B42] Sugiyama Y Watase Y Nagase M Makita N Yagura S Hirai A Sugiura M The complete nucleotide sequence and multipartite organization of the tobacco mitochondrial genome: comparative analysis of mitochondrial genomes in higher plants Mol Genet Genomics 2005 272 6 603 615 10.1007/s00438-004-1075-8 15583938

[B43] Alverson AJ Wei XX Rice DW Stern DB Barry K Palmer JD Insights into the evolution of mitochondrial genome size from complete sequences of *Citrullus lanatus *and *Cucurbita pepo *(Cucurbitaceae) Mol Biol Evol 2010 27 6 1436 1448 10.1093/molbev/msq029 20118192 PMC2877997

[B44] Kent WJ BLAT - The BLAST-like alignment tool Genome Res 2002 12 4 656 664 11932250 10.1101/gr.229202 PMC187518

[B45] Knaus B Short read toolbox http://brianknaus.com

[B46] Gordon A FASTX-Toolkit http://hannonlab.cshl.edu/fastx_toolkit/index.html

[B47] Li H Durbin R Fast and accurate short read alignment with Burrows-Wheeler transform Bioinformatics 2009 25 14 1754 1760 10.1093/bioinformatics/btp324 19451168 PMC2705234

[B48] Nguyen P Ma J Pei D Obert C Cheng C Geiger T Identification of errors introduced during high throughput sequencing of the T cell receptor repertoire BMC Genomics 2011 12 1 106. 10.1186/1471-2164-12-106 21310087 PMC3045962

[B49] Gladman S Seeman T VelvetOptimiser version 2.1.7 http://bioinformatics.net.au/software.velvetoptimiser.shtml

[B50] Cantarel BL Korf I Robb SMC Parra G Ross E Moore B Holt C Alvarado AS Yandell M MAKER: An easy-to-use annotation pipeline designed for emerging model organism genomes Genome Res 2008 18 1 188 196 18025269 10.1101/gr.6743907 PMC2134774

[B51] Smit A Hubley R Green P RepeatMasker Open-3.0 http://www.repeatmasker.org

[B52] Jurka J Kapitonov VV Pavlicek A Klonowski P Kohany O Walichiewicz J Repbase update, a database of eukaryotic repetitive elements Cytogenet Genome Res 2005 110 1-4 462 467 10.1159/000084979 16093699

[B53] You FM Huo NX Gu YQ Luo MC Ma YQ Hane D Lazo GR Dvorak J Anderson OD BatchPrimer3: A high throughput web application for PCR and sequencing primer design BMC Bioinformatics 2008 9 253 10.1186/1471-2105-9-253 18510760 PMC2438325

[B54] He J Dai XB Zhao XC PLAN: a web platform for automating high-throughput BLAST searches and for managing and mining results BMC Bioinformatics 2007 8 53 10.1186/1471-2105-8-53 17291345 PMC1800871

[B55] Altschul SF Madden TL Schaffer AA Zhang JH Zhang Z Miller W Lipman DJ Gapped BLAST and PSI-BLAST: a new generation of protein database search programs Nucleic Acids Res 1997 25 17 3389 3402 10.1093/nar/25.17.3389 9254694 PMC146917

[B56] Hood GM PopTools version 3.2.3 http://www.poptools.org

[B57] Wu F Mueller LA Crouzillat D Petiard V Tanksley SD Combining bioinformatics and phylogenetics to identify large sets of single-copy orthologous genes (COSII) for comparative, evolutionary and systematic studies: a test case in the euasterid plant clade Genetics 2006 174 3 1407 1420 10.1534/genetics.106.062455 16951058 PMC1667096

[B58] Livshultz T Middleton DJ Endress ME Williams JK Phylogeny of Apocynoideae and the APSA clade (Apocynaceae s.l.) Ann Mo Bot Gard 2007 94 2 324 359 10.3417/0026-6493(2007)94[324:POAATA]2.0.CO;2

[B59] Angiosperm DNA C-values Database (Release 6.0, Oct. 2005) http://www.kew.org/cvalues

[B60] Meve U Species numbers and progress in Asclepiad taxonomy Kew Bull 2002 57 2 459 464 10.2307/4111126

[B61] Ravi V Khurana JP Tyagi AK Khurana P An update on chloroplast genomes Plant Syst Evol 2008 271 1-2 101 122 10.1007/s00606-007-0608-0

[B62] Samson N Bausher MG Lee SB Jansen RK Daniell H The complete nucleotide sequence of the coffee (*Coffea arabica *L.) chloroplast genome: organization and implications for biotechnology and phylogenetic relationships amongst angiosperms Plant Biotechnol J 2007 5 2 339 353 10.1111/j.1467-7652.2007.00245.x 17309688 PMC3473179

[B63] Jansen RK Wojciechowski MF Sanniyasi E Lee SB Daniell H Complete plastid genome sequence of the chickpea (*Cicer arietinum*) and the phylogenetic distribution of *rps*12 and *clp*P intron losses among legumes (Leguminosae) Mol Phylogenet Evol 2008 48 3 1204 1217 10.1016/j.ympev.2008.06.013 18638561 PMC2586962

[B64] Lee HL Jansen RK Chumley TW Kim KJ Gene relocations within chloroplast genomes of *Jasminum *and *Menodora *(Oleaceae) are due to multiple, overlapping inversions Mol Biol Evol 2007 24 5 1161 1180 10.1093/molbev/msm036 17329229

[B65] Guisinger MM Chumley TW Kuehl JV Boore JL Jansen RK Implications of the plastid genome sequence of *Typha *(Typhaceae, Poales) for understanding genome evolution in Poaceae J Mol Evol 2010 70 2 149 166 10.1007/s00239-009-9317-3 PMC2825539 20091301

[B66] Greiner S Wang X Rauwolf U Silber MV Mayer K Meurer J Haberer G Herrmann RG The complete nucleotide sequences of the five genetically distinct plastid genomes of *Oenothera*, subsection *Oenothera*: I. Sequence evaluation and plastome evolution Nucleic Acids Res 2008 36 7 2366 2378 10.1093/nar/gkn081 18299283 PMC2367718

[B67] Guisinger MM Kuehl JV Boore JL Jansen RK Extreme reconfiguration of plastid genomes in the angiosperm family Geraniaceae: Rearrangements, repeats, and codon usage Mol Biol Evol 2011 28 1 583 600 10.1093/molbev/msq229 20805190

[B68] Jansen RK Cai Z Raubeson LA Daniell H dePamphilis CW Leebens-Mack J Müller KF Guisinger-Bellian M Haberle RC Hansen AK Analysis of 81 genes from 64 plastid genomes resolves relationships in angiosperms and identifies genome-scale evolutionary patterns Proc Natl Acad Sci USA 2007 104 49 19369 19374 10.1073/pnas.0709121104 18048330 PMC2148296

[B69] Haberle RC Fourcade HM Boore JL Jansen RK Extensive rearrangements in the chloroplast genome of *Trachelium caeruleum *are associated with repeats and tRNA genes J Mol Evol 2008 66 4 350 361 10.1007/s00239-008-9086-4 18330485

[B70] Jo Y Park J Kim J Song W Hur C-G Lee Y-H Kang B-C Complete sequencing and comparative analyses of the pepper (*Capsicum annuum *L.) plastome revealed high frequency of tandem repeats and large insertion/deletions on pepper plastome Plant Cell Rep 2010 1 13 10.1007/s00299-010-0929-2 20978766

[B71] Cai ZQ Guisinger M Kim HG Ruck E Blazier JC McMurtry V Kuehl JV Boore J Jansen RK Extensive reorganization of the plastid genome of *Trifolium subterraneum *(Fabaceae) is associated with numerous repeated sequences and novel DNA insertions J Mol Evol 2008 67 6 696 704 10.1007/s00239-008-9180-7 19018585

[B72] Arthofer W Schuler S Steiner FM Schlick-Steiner BC Chloroplast DNA-based studies in molecular ecology may be compromised by nuclear-encoded plastid sequence Mol Ecol 2010 19 18 3853 3856 10.1111/j.1365-294X.2010.04787.x 20735742

[B73] Parks M Cronn R Liston A Increasing phylogenetic resolution at low taxonomic levels using massively parallel sequencing of chloroplast genomes BMC Biology 2009 7 84 10.1186/1741-7007-7-84 19954512 PMC2793254

[B74] Neubig K Whitten W Carlsward B Blanco M Endara L Williams N Moore M Phylogenetic utility of *ycf1 *in orchids: a plastid gene more variable than *matK* Plant Syst Evol 2009 277 1 75 84 10.1007/s00606-008-0105-0

[B75] Kuroda H Maliga P The plastid clpP1 protease gene is essential for plant development Nature 2003 425 6953 86 89 10.1038/nature01909 12955146

[B76] Drescher A Ruf S Calsa T Carrer H Bock R The two largest chloroplast genome-encoded open reading frames of higher plants are essential genes Plant J 2000 22 2 97 104 10.1046/j.1365-313x.2000.00722.x 10792825

[B77] Kode V Mudd EA Iamtham S Day A The tobacco plastid *accD *gene is essential and is required for leaf development Plant J 2005 44 2 237 244 10.1111/j.1365-313X.2005.02533.x 16212603

[B78] Konishi T Shinohara K Yamada K Sasaki Y Acetyl-CoA carboxylase in higher plants: Most plants other than Gramineae have both the prokaryotic and the eukaryotic forms of this enzyme Plant Cell Physiol 1996 37 2 117 122 8665091 10.1093/oxfordjournals.pcp.a028920

[B79] Gornicki P Faris J King I Podkowinski J Gill B Haselkorn R Plastid-localized acetyl-CoA carboxylase of bread wheat is encoded by a single gene on each of the three ancestral chromosome sets Proc Natl Acad Sci USA 1997 94 25 14179 14184 10.1073/pnas.94.25.14179 9391173 PMC28453

[B80] Kubo T Mikami T Organization and variation of angiosperm mitochondrial genome Physiol Plant 2007 129 1 6 13 10.1111/j.1399-3054.2006.00768.x

[B81] Knoop V The mitochondrial DNA of land plants: peculiarities in phylogenetic perspective Curr Genet 2004 46 3 123 139 15300404 10.1007/s00294-004-0522-8

[B82] Sanchez-Puerta MV Cho Y Mower JP Alverson AJ Palmer JD Frequent, phylogenetically local horizontal transfer of the *cox1 *group I intron in flowering plant mitochondria Mol Biol Evol 2008 25 8 1762 1777 10.1093/molbev/msn129 18524785 PMC2727383

[B83] Cusimano N Zhang L-B Renner SS Reevaluation of the *cox1 *group I intron in Araceae and angiosperms indicates a history dominated by loss rather than horizontal transfer Mol Biol Evol 2008 25 2 265 276 10.1093/molbev/msm241 18158323

[B84] Bennetzen JL The contributions of retroelements to plant genome organization, function and evolution Trends Microbiol 1996 4 9 347 353 10.1016/0966-842X(96)10042-1 8885169

[B85] Kumar A Bennetzen JL Plant retrotransposons Annu Rev Genet 1999 33 479 532 10.1146/annurev.genet.33.1.479 10690416

[B86] Vitte C Bennetzen JL Analysis of retrotransposon structural diversity uncovers properties and propensities in angiosperm genome evolution Proc Natl Acad Sci 2006 103 47 17638 17643 10.1073/pnas.0605618103 17101966 PMC1693799

[B87] Vitte C Panaud O LTR retrotransposons and flowering plant genome size: emergence of the increase/decrease model Cytogenet Genome Res 2005 110 1-4 91 107 10.1159/000084941 16093661

[B88] Cavagnaro P Chung S-M Szklarczyk M Grzebelus D Senalik D Atkins A Simon P Characterization of a deep-coverage carrot (*Daucus carota *L.) BAC library and initial analysis of BAC-end sequences Mol Genet Genomics 2009 281 3 273 288 10.1007/s00438-008-0411-9 19104839

[B89] Cavallini A Natali L Zuccolo A Giordani T Jurman I Ferrillo V Vitacolonna N Sarri V Cattonaro F Ceccarelli M Analysis of transposons and repeat composition of the sunflower (*Helianthus annuus *L.) genome Theor Appl Genet 2010 120 3 491 508 10.1007/s00122-009-1170-7 19826774

[B90] Datema E Mueller L Buels R Giovannoni J Visser R Stiekema W van Ham R Comparative BAC end sequence analysis of tomato and potato reveals overrepresentation of specific gene families in potato BMC Plant Biol 2008 8 1 34. 10.1186/1471-2229-8-34 18405374 PMC2324086

[B91] Whiteford N Haslam N Weber G Prugel-Bennett A Essex J Roach P Bradley M Neylon C An analysis of the feasibility of short read sequencing Nucl Acids Res 2005 33 19 e171. 16275781 10.1093/nar/gni170 PMC1278949

[B92] Rogers SO Bendich AJ Ribosomal-RNA genes in plants - Variability in copy number and in the intergenic spacer Plant Mol Biol 1987 9 5 509 520 10.1007/BF00015882 24277137

[B93] Sastri DC Hilu K Appels R Lagudah ES Playford J Baum BR An overview of evolution in plant 5S DNA Plant Syst Evol 1992 183 3 169 181 10.1007/BF00940801

[B94] Hillis DM Dixon MT Ribosomal DNA: Molecular evolution and phylogenetic inference Q Rev Biol 1991 66 4 411 453 10.1086/417338 1784710

[B95] Volkov R Kostishin S Ehrendorfer E Schweizer D Molecular organization and evolution of the external transcribed rDNA spacer region in two diploid relatives of *Nicotiana tabacum *(Solanaceae) Plant Syst Evol 1996 201 1-4 117 129 10.1007/BF00989055

[B96] Borisjuk NV Davidjuk YM Kostishin SS Miroshnichenco GP Velasco R Hemleben V Volkov RA Structural analysis of rDNA in the genus *Nicotiana* Plant Mol Biol 1997 35 5 655 660 10.1023/A:1005856618898 9349286

[B97] Bena G Jubier M-F Olivieri I Lejeune B Ribosomal external and internal transcribed spacers: Combined use in the phylogenetic analysis of *Medicago *(Leguminosae) J Mol Evol 1998 46 3 299 306 10.1007/PL00006306 9502673

[B98] Volkov RA Komarova NY Panchuk II Hemleben V Molecular evolution of rDNA external transcribed spacer and phylogeny of sect. *Petota *(genus *Solanum*) Mol Phylogenet Evol 2003 29 2 187 202 10.1016/S1055-7903(03)00092-7 13678676

[B99] Maggini F Gelati MT Spolverini M Frediani M The intergenic spacer region of the rDNA in *Olea europaea *L Tree Genet Genom 2008 4 2 293 298 10.1007/s11295-007-0109-x

[B100] Cronn RC Zhao XP Paterson AH Wendel JF Polymorphism and concerted evolution in a tandemly repeated gene family: 5S ribosomal DNA in diploid and allopolyploid cottons J Mol Evol 1996 42 6 685 705 10.1007/BF02338802 8662014

[B101] Fujiwara M Inafuku J Takeda A Watanabe A Fujiwara A Kohno S Kubota S Molecular organization of 5S rDNA in bitterlings (Cyprinidae) Genetica 2009 135 3 355 365 10.1007/s10709-008-9294-2 18648989

[B102] Saini A Jawali N Molecular evolution of 5S rDNA region in *Vigna *subgenus *Ceratotropis *and its phylogenetic implications Plant Syst Evol 2009 280 3-4 187 206 10.1007/s00606-009-0178-4

[B103] Stage DE Eickbush TH Sequence variation within the rRNA gene loci of 12 *Drosophila *species Genome Res 2007 17 12 000. 10.1101/gr.6376807 PMC2099596 17989256

[B104] Ganley ARD Kobayashi T Highly efficient concerted evolution in the ribosomal DNA repeats: Total rDNA repeat variation revealed by whole-genome shotgun sequence data Genome Res 2007 17 2 184 191 10.1101/gr.5457707 17200233 PMC1781350

[B105] Kolosha VO Fodor I Nucleotide sequence of *Citrus limon *26S ribosomal-RNA gene and secondary structure model of its RNA Plant Mol Biol 1990 14 2 147 161 10.1007/BF00018556 2101688

[B106] Nei M Rooney AP Concerted and birth-and-death evolution of multigene families* Annu Rev Genet 2005 39 1 121 152 10.1146/annurev.genet.39.073003.112240 16285855 PMC1464479

[B107] Baxter I Tchieu J Sussman MR Boutry M Palmgren MG Gribskov M Harper JF Axelsen KB Genomic comparison of P-Type ATPase ion pumps in *Arabidopsis *and Rice Plant Physiol 2003 132 2 618 628 10.1104/pp.103.021923 12805592 PMC167002

[B108] Thomson RC Wang IJ Johnson JR Genome-enabled development of DNA markers for ecology, evolution and conservation Mol Ecol 2010 19 11 2184 2195 10.1111/j.1365-294X.2010.04650.x 20465588

