# Supplementary material for: Building a model: developing genomic resources for common milkweed (Asclepias syriaca) with low coverage genome sequencing
Source: BMC Genomics. 2011 May 4;12:211. doi: 10.1186/1471-2164-12-211 (PMC3116503; doi:10.1186/1471-2164-12-211)

Additional file 1: Polymorphism detected among Illumina reads for the PhiX sequencing control
The blue line represents the conservative 2% cut-off used to differentiate between sequencing error and polymorphism for *Asclepias syriaca*.

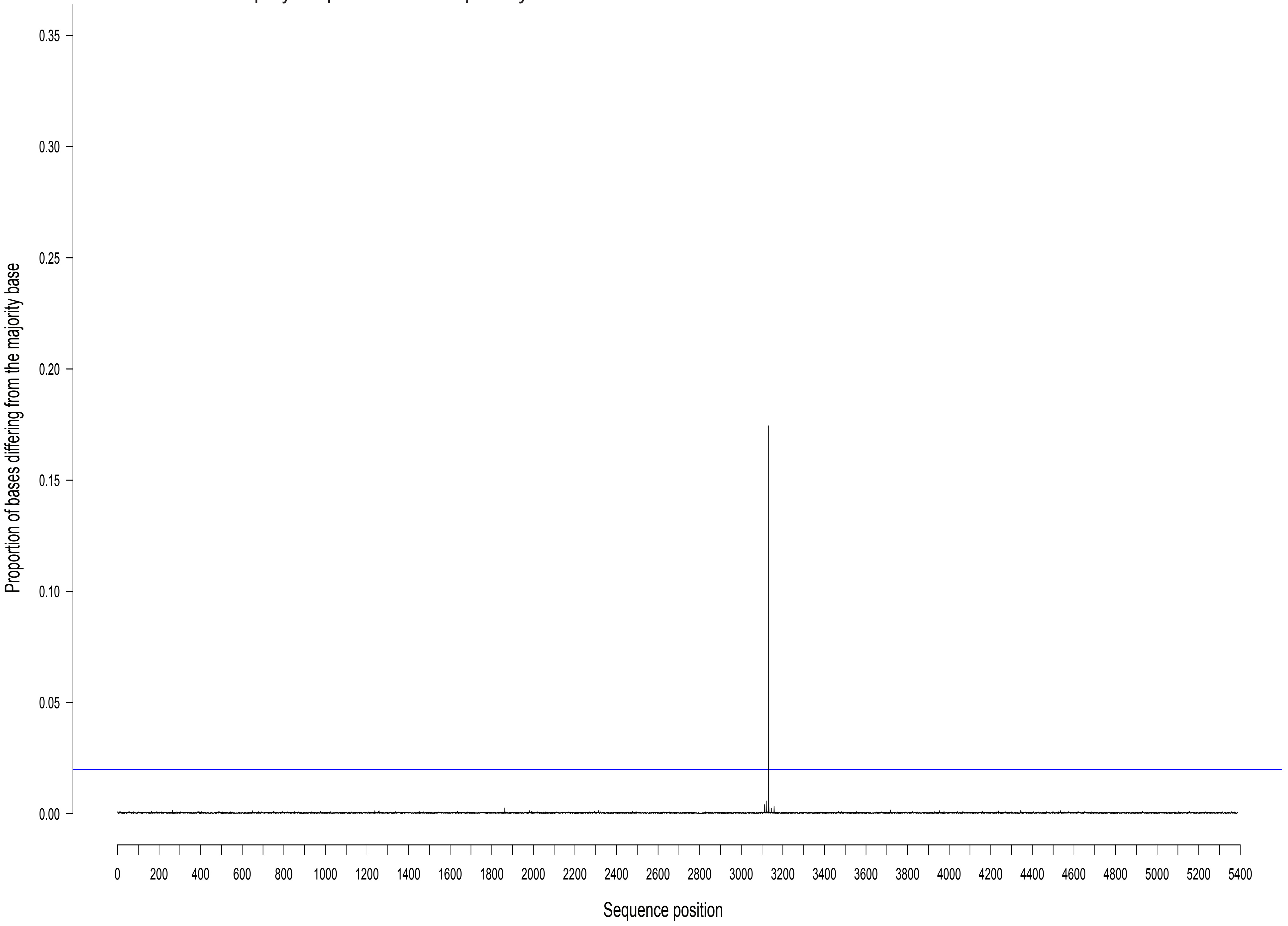

Supplement: Additional file 1 — Polymorphism detected among Illumina reads for the PhiX sequencing control. A graph showing the proportion of bases differing from the majority base in the Illumina PhiX sequencing control. [file 1471-2164-12-211-S1.PDF]
