# Supplementary material for: Building a model: developing genomic resources for common milkweed (Asclepias syriaca) with low coverage genome sequencing
Source: BMC Genomics. 2011 May 4;12:211. doi: 10.1186/1471-2164-12-211 (PMC3116503; doi:10.1186/1471-2164-12-211)
Supplement: Additional file 2 — Primer sets designed using BatchPrimer3 for 184 nuclear microsatellite loci in Asclepias syriaca not tested for amplification success. A table of nuclear microsatellite locus primer sets, including predicted product size and repeat motif information. [file 1471-2164-12-211-S2.PDF]

Additional file 2 – Primer sets designed using BatchPrimer3 for 184 nuclear microsatellite loci in *Asclepias syriaca* not tested for amplification success

See Table 2 for 25 successfully amplified additional loci. Primer names with identical numbers followed by letters indicate multiple microsatellites originating from the same contig.

Primer Set Name	Orientation	Primer Sequence (5'-3')	Tm (°C)	Repeat Motif	Product Size (bp)
As121	FORWARD	GTCGTCTTCCCCTTTTTC	54.6	$AAAG_3$	114
	REVERSE	GTTCTATTCGAATTTCGTTCC	55.5		
As370	FORWARD	CCTCACTTTAAGTTGGTGGA	55.3	CT_4	100
	REVERSE	TTATCGAGTCGAATGAGATGT	54.9		
As525	FORWARD	TGGAAGGAAGGTAGTAAGGTC	55.1	TA_4	109
	REVERSE	AGATTCCCTTGAAGCTAGAGA	55.0		
As738	FORWARD	AATGGCATAGAGGCATATTTA	54.2	AGC_4	113
	REVERSE	CTATGCTATCAAGAAGCAGGA	55.0		
As810	FORWARD	TAGATGATAGCACCAATCCAC	55.2	$GCTT_3$	104
	REVERSE	AGGACAAGATAAGATGGCCTA	55.6		
As1220	FORWARD	TTGGGAATCAACTGAACTAGA	55.0	CT_4	105
	REVERSE	CCGGGAGAAAAGTGATCT	55.1		
As1274	FORWARD	GTAAAGTGGCATTGAGGAGAT	55.9	AG_4	100
	REVERSE	AAACGTGACTGGGTCATCTA	55.6		
As1380	FORWARD	CTGTTGATTGTTTGGACAGAT	55.1	AG_4	116
	REVERSE	AACTAAATGCACGGTAAGATG	54.6		
As1678	FORWARD	CACCCCTATACGGATAACTTC	55.3	AT_4	104
	REVERSE	TCTTTCTGTATATGGCAAAACTT	55.1		
As1925	FORWARD	GACATCGATTTTGCACATATAC	54.8	$ATCG_3$	120
	REVERSE	TACTCACCTCAACAAAATGGT	54.7		
As2003b	FORWARD	AGCCCCTTTATTAAGTGATTG	55.2	$AATC_3$	110
	REVERSE	TAGGCGATTTAAGCTTTATGA	54.7		
As2159	FORWARD	AGCTTAGCTTGAGGGTTAAAG	54.9	TA_4	123
	REVERSE	ACTATTGAAAGAAAGACAGGTG	53.0		
As2819	FORWARD	AAGCTCCTACACCTCCATATC	55.0	TC_4	180
	REVERSE	TCTCTTATTTATGGAACTTCTTC	51.7		
As5408	FORWARD	AGTTAAGAAGAAGTGGGTTCG	55.3	CT_4	115
	REVERSE	GAAATGCCAATTCAACTCTCT	56.0		
As5755	FORWARD	TCTCAGCTAGACTCAGAAGGA	54.5	$GTTAG_3$	102
	REVERSE	GAAAACTACCAAGACTGACGA	54.6		
As5757	FORWARD	TTGAAGCTACCCGTAGATATG	54.7	AG_4	114
	REVERSE	AACCTCTTTAGTTTTGCCAAC	55.3		
As5836	FORWARD	GAATGTCGTCCTTTCATTGT	55.0	AG_4	114
	REVERSE	CGAATAGGAAGGTATGGAAGT	55.0		

As6143a	FORWARD	AAGAAGTGCAACCATATATCAA	54.2	TA_4	122
	REVERSE	GTTGTAAATTTCCTGCATTTG	55.0		
As6143b	FORWARD	AAGAAGTGCAACCATATATCAA	54.2	AT_4	122
	REVERSE	GTTGTAAATTTCCTGCATTTG	55.0		
As6929a	FORWARD	GAGTGTTTGAAGAGGGATTCT	55.0	GA_4	121
	REVERSE	TTCTTACTACTTTCCCATAGCC	54.6		
As6929b	FORWARD	CGGCTATGGGAAAGTAGTAAG	55.9	AT_4	121
	REVERSE	TAGGGGGAAAATCGAGTATAG	55.2		
As6942	FORWARD	TGGAATAGGTGTCTTCAACTG	55.3	TC_4	111
	REVERSE	TTGGTCGGTCTAAAGATAACA	55.0		
As7703	FORWARD	AGGCTAAGCTCTCAACCTAAC	54.8	TA_4	108
	REVERSE	CTTCAAAGCAAAGAGGGTAA	54.9		
As8174	FORWARD	AGAATGCTTCTTGCTAACATC	54.0	$GCAA_3$	112
	REVERSE	CCTTAGAAAGCACAGACTGC	55.4		
As8578	FORWARD	GCCCTGACGATATAAGTTTTT	55.2	$TCTT_3$	103
	REVERSE	GTGGCTCCATACCTCAGAT	54.9		
As8868	FORWARD	AAAGAAGATGCTGACACAAAG	54.8	AG_4	101
	REVERSE	GACTTAGCTGACTACCATTCG	54.3		
As8870	FORWARD	GCAGCTCCTTCTATGCTTATT	55.6	GA_4	122
	REVERSE	CACCATAATCGATTTCACATT	55.0		
As9296a	FORWARD	TAAGTGGATCGTTTCTTCAAA	55.1	TA_4	100
	REVERSE	AGGCTTCTGCTAAAAAGAAAA	55.4		
As9296b	FORWARD	GAAGCAACATTAAGGCCTATC	55.7	AGA_4	102
	REVERSE	AGGAAAGGTTGTTAGAAAACTG	54.5		
As9296c	FORWARD	GTTCCCTCCACTTATTAGAGC	54.8	TCT_4	119
	REVERSE	GCTTCAACGTAAAGGATAAGG	55.8		
As9296d	FORWARD	TATCTAAGCTCGGCTAGAAGC	55.9	$GGATA_3$	106
	REVERSE	TAGAAAACTGGGCCTTACTCT	55.0		
As9299	FORWARD	GTAGCAAAGCGAATGAACA	55.0	AT_4	103
	REVERSE	TGATTAGGAACGATACGAGAA	55.1		
As9388	FORWARD	TTTTGAGAAACTACCGTTGAG	54.8	AT_4	108
	REVERSE	AATGCATACCTTTATGATGCT	54.1		
As9478	FORWARD	GGCATCAAAATTCTCTAAACA	54.7	CAT_4	113
	REVERSE	AGATCGAAGAAGACTGTGATG	54.5		
As10870a	FORWARD	TAGATTTTTCCGAAGACTGAG	54.0	GA_4	100
	REVERSE	TACCAATAGGATTCTCTGCAA	55.1		
As10870b	FORWARD	ACGAAGAGCAGGAGGTTAGTA	55.9	$AAGA_3$	101
	REVERSE	CTGACTCCAATACAAATACCG	54.9		
As11441	FORWARD	AGCAAATAATGCCAAAAATC	54.6	AAG_4	100
	REVERSE	CAAGTACCGGGTAGGTAAGAT	55.0		
As11689a	FORWARD	CCCGAGGGAAATTAATAGATA	55.1	TC_4	121
	REVERSE	ATGAATGACCCATAGTACGC	55.0		
As11689b	FORWARD	TTTTGTATTTGCCAACCTAGA	55.2	AG_4	107

	REVERSE	ACGGGTGAAATCCTATATTTT	54.5		
As11689c	FORWARD	GTTGCTTTGGACAGGTAAGAT	56.0	AG_4	102
	REVERSE	GGGTAAGTTCCCAAGCAT	55.0		
As12120	FORWARD	GGCATTCCTTTATCTTCTAGC	55.0	$TTCT_3$	107
	REVERSE	CACCAACTAACAGTCGGATAC	54.8		
As13014	FORWARD	CAAGAAGCGACTCAAACTCTA	55.1	AG_4	131
	REVERSE	CCTATCCAGTCCAGAGAAAAT	55.0		
As13124	FORWARD	GCTTATGGTAAAGCTTCCTGT	55.4	CT_4	100
	REVERSE	AAGAAAGAAAAGAGAGAGTTGC	54.1		
As13403	FORWARD	GGGAGAAATTCCCTATCTAAA	54.2	AG_4	110
	REVERSE	ATCTGTGGTACTGAAGTCGAG	54.4		
As13641	FORWARD	GAAGTCAAGCAGTTCGACTAA	54.9	CT_4	103
	REVERSE	GAAAACAAATTAGGAAAGAAGTC	53.5		
As14767	FORWARD	ACTAGTACCTCCCAGTGAAGC	55.2	CT_4	106
	REVERSE	CACCCTTATCAAAACTTCCTT	55.1		
As14821	FORWARD	CCTTATTGCAGGAAAACACTA	54.8	AT_4	116
	REVERSE	AACTACATTTATGCGAATACGG	56.1		
As14892a	FORWARD	ACCAGATGAGATGATGTTGAG	55.0	$TTCT_3$	109
	REVERSE	AGGGAACTCCACTAATTTATGA	54.7		
As14892b	FORWARD	ACCAGATGAGATGATGTTGAG	55.0	$CAATT_3$	109
	REVERSE	AGGGAACTCCACTAATTTATGA	54.7		
As15779	FORWARD	AGCCAAGCAAAATAAAAAGAG	55.7	GA_4	133
	REVERSE	AATCGAGATTGGGTCAGTATT	55.3		
As15913	FORWARD	TTTTACCGAAGCAATACAAAG	55.0	TGA_4	118
	REVERSE	ACTTCATTTACGGCATTGATA	55.0		
As16072	FORWARD	GATTCCATCCAGAAGAATGAT	55.6	AAG_4	120
	REVERSE	GTATCTTTCTACCTCTCCTTGG	54.0		
As16238a	FORWARD	TCATCTCAAATCAAACTACCG	55.4	TC_4	128
	REVERSE	CACCATCCCTACCTTGATAGT	55.6		
As16238b	FORWARD	AGTGGCGAAGTGTTGATTAC	55.3	GAA_4	120
	REVERSE	TTGATTTGAGATGAGAGGCTA	55.2		
As16392	FORWARD	CTATTGGCTTCACCAGTACC	54.8	CT_4	106
	REVERSE	GCAGTGAGAAGGATATCATCA	55.3		
As16526	FORWARD	AAGAAGCACACCAGACTAGAA	54.3	$GCAA_3$	108
	REVERSE	AGAGGCGAACATTTCTCTATT	54.9		
As18901	FORWARD	AAATAGCCAGATTGGGATAAG	55.1	CA_4	110
	REVERSE	ACTGCTATTTCAACTGATGGA	55.0		
As19495	FORWARD	GTGTTGAACTGGATGACATTT	55.0	AG_4	104
	REVERSE	GCGGTCTCACTTTTTCTAATA	54.0		
As19903	FORWARD	AATTAGGTCAAGGGATGCTAC	55.0	GA_4	113
	REVERSE	CATTTGGGATATTTGAATCAC	54.5		
As20020	FORWARD	TCGTTTCAAGAAAGAGTTTTG	54.9	$TCTT_3$	122
	REVERSE	GTAAGTTAGCGGCGTAATAAG	54.0		

As20679b	FORWARD	AATGAAGGTTGAGTTGAATGA	54.8	$TTCG_3$	104
	REVERSE	CGAGAAGAGATAAAAGCAGAA	54.3		
As20758	FORWARD	CCTTTAACCTTTAACCGAGAA	55.4	AT_4	119
	REVERSE	GGAAGGACCAGAGAATTTAGA	55.1		
As20767	FORWARD	TCTTTCTGATGAGACTTTTGC	54.8	$AGAA_3$	114
	REVERSE	AGTAGGAGCCTTTTCTACCAA	55.0		
As21133	FORWARD	CCGCAGTTTTTCTTGATTAT	54.7	CT_4	109
	REVERSE	CCTCTTGGTCTACAGCCTTAT	55.3		
As21149a	FORWARD	ATACATTCTCCGTGCCTTTA	54.9	CT_4	122
	REVERSE	TGAACTAATTCCTTCTTGTGC	54.7		
As21149b	FORWARD	GGCATATCTTTGTGCCTACTA	54.7	AG_4	144
	REVERSE	GGAACTGATGACCTTTCTTCT	55.0		
As21649	FORWARD	AACCATCCTCCTTATGTCACT	55.2	AC_4	101
	REVERSE	AGTGTAAGGACAAGAAGCACA	55.1		
As22120	FORWARD	CCACTCGACTGTAAGGATGTA	55.4	TG_4	102
	REVERSE	GCACTGTCCCAAAGAGTAAT	54.4		
As22167	FORWARD	TGCTGTATGAATTTGAAGAGTC	54.7	TA_4	130
	REVERSE	ACCAATTTGATAACAAGAGCA	55.0		
As22376	FORWARD	AAAGTGCGCTTATAAATCGTAT	55.2	AT_4	100
	REVERSE	GGTGGTATTCTCAGGGATAAC	55.1		
As22687	FORWARD	CGATATTCAACGACTAACGAT	54.6	$TAAC_3$	105
	REVERSE	CCTTCCATTTTACAGAGGTTT	55.1		
As22763	FORWARD	ATGATAGGTTTGAGCCGATT	56.3	AC_4	101
	REVERSE	GTTGCTCCTTCTACCCTTAAC	54.8		
As23491b	FORWARD	TCTCTGACTACCCACAAGATG	55.3	CTA_4	103
	REVERSE	GGAGTTTTGTGACATTGAAGA	55.3		
As24305	FORWARD	GGAGCCTGATGTACAACTTAG	54.2	GA_4	137
	REVERSE	CATCTGCACTGTGATGAGTC	55.0		
As24519	FORWARD	CCAATAAACAAAGAGAAAGCA	54.9	AG_4	112
	REVERSE	GCCTTACGTAGATGTAGTGGA	54.6		
As25019	FORWARD	AATCATTCATTTCCTCTATCGT	54.6	TA_4	100
	REVERSE	CAAACAAATATGGCAATAAGG	55.0		
As25105	FORWARD	GCTTGCTTACTTCATTTTACG	54.7	$ATTC_3$	104
	REVERSE	CTAGCCCATACCCTTTGAGT	55.9		
As25487	FORWARD	ACGCAACTACATATGTCCATC	55.1	CT_4	135
	REVERSE	ACAATAGCGAGAAAGGAAAAC	55.4		
As27644	FORWARD	AGAGAGAGAGAGATGAGGAAGA	54.1	AT_4	106
	REVERSE	TTTTCGTCCCTAAACTTTGTT	55.7		
As27669	FORWARD	CGAATCATATGTTGTCGATAG	53.3	AT_4	105
	REVERSE	ACAGCGCTTACTCATCAAAG	55.8		
As28083	FORWARD	AAACATCAACGGTAGTGTCAT	54.6	CA_4	107
	REVERSE	AGAGGAGAATGGTCAGACTTC	55.0		
As28858	FORWARD	AGGTCAGGGTATTGGTAGAAA	55.4	CT_4	111

	REVERSE	ATCCATATCGACTTCCAAGAG	55.8		
As29134	FORWARD	TTGAGAACTGAACTGAGAAGC	54.9	TC_4	111
	REVERSE	CCGTAGATGGATGAATCATAA	55.1		
As30342	FORWARD	AAGGATCTAACTTCAACTTTCG	54.6	TA_4	106
	REVERSE	CTCTTTGCGTGGATAATCTAA	54.9		
As30511	FORWARD	GACCTGATCCCCTTATAGAGA	54.9	AG_4	111
	REVERSE	GGTTCGATCTGACATCTTTACT	55.1		
As33389	FORWARD	AGGGGATAAGAGAACTGGATT	55.9	AG_4	109
	REVERSE	GGGACTACCCAGCTACTTCT	55.1		
As34183	FORWARD	TTTAAAGTTTTAGTGGGCAAG	54.2	GT_4	101
	REVERSE	CGTGCACTCCTTTTAGATTC	55.1		
As34695	FORWARD	ATGAAAGAGAAGAAAGAGTGAGA	54.3	TC_4	100
	REVERSE	GGGTTTACAGTTTCCAAATTC	55.3		
As35600	FORWARD	CCCTCTTTTCCAACTAGAACT	54.4	GA_4	116
	REVERSE	AGGAATTAGGCTTAGAAGACG	54.7		
As36198	FORWARD	TCGAAGAGCTAGATGTAATGG	54.8	GA_4	107
	REVERSE	ATAGGTTGGAGTTCCTGTAGC	55.1		
As37690	FORWARD	GCTCTTCTAGCTTTGGATCTT	54.8	$CCAATA_3$	170
	REVERSE	GTATTTATCGAAAGACGAGCA	54.8		
As38257	FORWARD	GACCTTAACTCGGTCTTGACT	55.1	CT_4	100
	REVERSE	TGTCTTGTGAAAAGAAACTCA	53.6		
As40666	FORWARD	TCTTCCATTGATCAACCATAC	55.0	GA_4	130
	REVERSE	GCTGACGTCATGGATTATTT	55.2		
As40723	FORWARD	AAGTAATCGACGGAGTGTGTA	54.9	AC_4	100
	REVERSE	ACGTTCGACACTTGGAACT	55.6		
As41059	FORWARD	GAACACAAACCAATGGTAGAG	54.7	AG_4	159
	REVERSE	AGGTATTCCTGTCTCCTTCAG	55.1		
As42098a	FORWARD	GAATCATGTCTGGATTTAGACTC	54.6	TA_4	122
	REVERSE	CTCTGGTTGGCTTATATGGTA	54.6		
As42098b	FORWARD	GCCACTCACTATCCACTATCA	55.3	CT_4	101
	REVERSE	AAGCCGTAGTTCTAGTTAGGG	54.7		
As42675	FORWARD	CCTGAGGATCAAATTCCTTAT	54.9	TA_4	100
	REVERSE	GGGTATAGCTCATAGCTCAAAT	54.5		
As42784	FORWARD	ATCGTCGATGATCGGTATAAT	56.1	AT_4	103
	REVERSE	CCAGGCATTAAGCTATAGACA	54.9		
As45012	FORWARD	CTGGGACAACAATAAAATCAA	55.3	$TGAT_3$	109
	REVERSE	CAGCGTGCTTTTATGAACTAT	55.0		
As46803	FORWARD	GGATCAGGTAAGGTAAACGTAA	55.2	TA_4	133
	REVERSE	TCTCAATCTTTCAATTCATGG	55.3		
As50236	FORWARD	AGGAGGAAAGAAACATCCAT	55.2	CA_4	116
	REVERSE	ATGGTGATTATGGACTCAGC	55.0		
As53582	FORWARD	ACACTCTTCATCAGCTTTTCA	55.2	TC_4	108
	REVERSE	GTTAGAGAAGGTGGTTGCTCT	55.3		

As54423	FORWARD	GCTTTAATGGTTATGTGAAAGA	53.9	AT_4	118
	REVERSE	AACAAGATGAAATTTAGGGTTC	54.3		
As55542	FORWARD	ACCCAATCAATTAACCCTAGA	55.3	$ATGC_3$	107
	REVERSE	GTGAAAGAACTTAAGGCAATG	54.3		
As55715	FORWARD	GCAAGTTTTTGCTCTTGATT	54.8	$TTTCA_3$	107
	REVERSE	AAACCTGTACATGGAACAAAA	54.8		
As55919	FORWARD	ATCAATTCATGATGCGAAAT	55.5	AT_4	101
	REVERSE	TTCCTTTTCTTGGAACAATTC	56.0		
As57343	FORWARD	CGTTAGCACTTTTGACTTGAC	55.3	CT_4	106
	REVERSE	GACTGCTTATTCCGCTAAAAC	55.9		
As57505	FORWARD	TAAGAAGTGCCCTGCATACT	55.2	TA_4	134
	REVERSE	AAGAAGCTTTGGCTTATTTTC	54.8		
As58725	FORWARD	TAGATAGACTTCTGCCAGCAC	54.9	TA_4	105
	REVERSE	ATTCCGGTCTCACAAGAAA	55.6		
As59892a	FORWARD	AGTGTACAGGCTACGGTACG	55.5	AG_4	152
	REVERSE	ACATAGCCATAGCCGAATTA	55.1		
As59892b	FORWARD	CAAGAAGAGACAACACTCTGG	55.1	$TACGG_3$	133
	REVERSE	GTCAAGCTGCTCCTCTCTC	55.2		
As60548	FORWARD	CGTTCATCCTTAATTTTCTTC	53.4	$TTTC_3$	148
	REVERSE	ATGATGAACAGGAAAGGAAAC	55.7		
As61119	FORWARD	ATCATGCTCAGAAAATAATGC	54.6	AT_4	113
	REVERSE	TGCTACCATGTACTCTAGGTTT	53.5		
As63797	FORWARD	GAATAGATAACCCCTCAGCAC	55.4	GA_4	133
	REVERSE	GTCTCATCCGTATCATTTCAA	55.2		
As64039	FORWARD	ACTTTCCATTGCAATCATCTA	54.9	$CATT_3$	111
	REVERSE	CTGAATGGATGATGAACTTTT	54.3		
As64726	FORWARD	TGATTAATTTGTTGTGGCTCT	55.0	$TTTC_3$	107
	REVERSE	ACCGAACTACAGAGTCGTACA	55.0		
As65701	FORWARD	TGTAAACGAACAACTGTACATAA	52.9	AT_4	108
	REVERSE	CCACCACTCTACTACCTTCGT	55.9		
As65986	FORWARD	TGCAGTAGCCTACAGTAGTGA	53.9	AG_4	114
	REVERSE	GTCTCTAGTCGAGATGGGAAT	55.0		
As66599	FORWARD	GAGACTAAGAGGTGCCTGATT	55.2	AG_4	144
	REVERSE	TGAACCAAACTATCTTACCTCA	54.2		
As66787	FORWARD	TATAGATGGCACGTATTACCC	54.4	GA_4	113
	REVERSE	CATACGGTCAGAAAGCTCAG	56.1		
As67765	FORWARD	ATGATGTTGTGTTCGAAGAAG	55.3	AC_4	121
	REVERSE	ACTCCTTGTTCTTCTCCACTC	55.1		
As68506	FORWARD	TGATTCATCATCCTCTTTGTT	54.7	AG_4	100
	REVERSE	CCAATGGATATCACATGAAGT	54.9		
As69254	FORWARD	GAATGGGTATTTAGGCTCTGT	55.0	TG_4	107
	REVERSE	AAATCCAGTTAAAACCACCAG	55.8		
As70080	FORWARD	CCTGCAACTATTCCTCTCTTT	55.4	TCT_4	106

	REVERSE	TTGTAACAAAAGGTGATCCTG	55.3		
As71066	FORWARD	GTTGACTCGCTTAAAAGAACA	55.0	CT_4	102
	REVERSE	CCTAGGAAACATAACAAGGAC	53.1		
As72624	FORWARD	CATAATCCTCAAAGTCCAAGTT	55.1	AAC_4	109
	REVERSE	ATGTATGAAGTTGAGGTGCTT	54.0		
As74249	FORWARD	ATAACTTGTTGACTGCAAAGC	54.8	AG_4	102
	REVERSE	GCACTTCAGCAGTAGATTCAA	55.8		
As74784	FORWARD	GTGAATGCATGGATTAAAGAA	55.3	CCA_4	102
	REVERSE	AATAGTGAAACCAAACGGAAG	56.0		
As77526	FORWARD	TTTCCTAATATCTGCTTGCTG	55.0	AC_4	128
	REVERSE	GCACAGTAGGCTGTAGTCATT	54.7		
As80042	FORWARD	CTTGCATCATTTCATTATCCT	54.4	TA_4	100
	REVERSE	ATAAATGTGCACTTGGTGCTA	56.0		
As82870	FORWARD	GGTTGGTTTATCATTTCTGCT	55.9	ATT_4	123
	REVERSE	TTGTCACATTGAATGAAGC	52.7		
As86065	FORWARD	TCTTCAATATAACCATCATTCG	54.5	TA_4	104
	REVERSE	TAGATGCAGATAGGCAAAAAC	54.8		
As88477	FORWARD	GTTTGTTGAGGGAAATTATCA	54.3	TTG_4	100
	REVERSE	CAATGCAACAATTTCTCAATC	55.8		
As91992	FORWARD	AAAGTCTTGGAGTTGAACCA	54.8	AT_4	120
	REVERSE	TCTCTAGGTCAAGCACTGAAG	54.9		
As92441	FORWARD	TAGGCAGAATACAAAGATGGA	55.1	AG_4	103
	REVERSE	CTCAACACTATTCCAGTGCAT	55.3		
As93437	FORWARD	CTTTCAACCAAATAGCTTTCA	54.9	$CTTTT_3$	104
	REVERSE	ATAAACATGCTAACCCGAGA	54.9		
As95613	FORWARD	TTTCTTTTCCTCTAACCACAA	54.3	TA_4	112
	REVERSE	GTTCTGCTCCTTTGAATGTAA	54.7		
As97041	FORWARD	TCAAAGAAACAAGAATTGCAC	55.6	$CAAA_3$	129
	REVERSE	AGGTTAGTATGGCTTGTTTCC	55.1		
As97117	FORWARD	CCGGGTTCTCATCTTTTATC	56.2	TA_4	100
	REVERSE	GCTAGAGCAAGTTTTGCTACC	56.2		
As97343	FORWARD	GATTCAATCCGTTTGATAACTT	55.0	TA_4	100
	REVERSE	TAACGTTTCTTCATGCATAGC	55.7		
As98879	FORWARD	AGACATGCGAATGGATCTT	55.6	GA_4	100
	REVERSE	TGCACACTCCAACCTAGAGTA	56.0		
As99992	FORWARD	TGAGCCAAAAAGAGTAAGAGT	53.6	GT_4	105
	REVERSE	GTCTTCACAATCAAATCTTCG	54.9		
As102617	FORWARD	CAGTGAGGAGGCAGAAAAC	55.9	CT_4	109
	REVERSE	GAGGAGCTTCAGTATCAAGG	54.1		
As109460	FORWARD	GGACTCTTGAATTTGGTCTTC	55.4	TC_4	121
	REVERSE	TAAACACAATAACCCGAAGTG	55.4		
As110213	FORWARD	TTATCAAAAGCAACCTCAAAG	54.9	GAT_4	109
	REVERSE	GCTCTAATCGATCCAATATCC	55.4		

As110592	FORWARD	AATGGACATGAATGTTTTGAG	55.1	GA_4	105
	REVERSE	AAGCAGAGGTGACAGTGATAG	54.6		
As110792	FORWARD	GTTCTTTCTATGCCGATCTTT	55.3	TG_4	114
	REVERSE	CAACCTAGAGACAAACCAGAA	54.6		
As111193	FORWARD	TCCTCCAAATTTCAATTCTT	53.9	TCA_4	116
	REVERSE	TGTGGAATCAGTTGTACCTTT	54.7		
As111807	FORWARD	GAGATACCTCGAACATCATCA	55.1	AGA_4	102
	REVERSE	TGACTCTGCTCTCTTTATTCG	55.0		
As116994	FORWARD	ATACACAAGCAGCAGAATCAT	55.0	CAG_4	101
	REVERSE	TGAAACCGACAACTATTTCAT	54.8		
As119055	FORWARD	AACACACACACAAACAAAACA	54.9	GAA_4	104
	REVERSE	CCTCTGTTATGTCAACACTCG	55.9		
As119087	FORWARD	TTTCTGTTTCACTATGGAGGA	55.0	$GAAG_3$	101
	REVERSE	CACATACGTCATCATTCAAAA	54.6		
As120404	FORWARD	CAAACAATGAAAAAGTGTCGT	55.4	AG_4	103
	REVERSE	CTTTGAAGGATCCGAGTGTAT	55.9		
As123452	FORWARD	AGGTAAGGATGAAAACGAAGT	54.7	$GAAG_3$	110
	REVERSE	GGTCTTTCAATGCTTTAATCTT	54.6		
As125868	FORWARD	AACTTCCCCAAGACTAATGAA	55.5	$CAAC_3$	116
	REVERSE	ATTGTGTTCAATTGATCTGCT	54.8		
As126609	FORWARD	AAAGCAGAGTATGACCATTGA	55.0	TG_4	137
	REVERSE	AAAATACCGATCAATCCTCTC	54.9		
As129320	FORWARD	TCTCAATGACAACCTACTGGT	54.7	CT_4	101
	REVERSE	TTTCTTCACTTCAGCTTGCTA	55.7		
As135378	FORWARD	AGTAGGAGTCATTTCCTGACC	54.9	TA_4	118
	REVERSE	TATCTTTAATGGCATAGTTCG	52.5		
As136405	FORWARD	CATTGAGAGAATCTGAGTTCG	55.1	$TTAA_3$	131
	REVERSE	CTCAGTTCTTTAGCTCGTTGA	55.1		
As138896	FORWARD	TTTCACAATCTCTTCCCATC	55.1	$TTCC_3$	124
	REVERSE	CCCACTATTTATGATAGAGATAGAA	53.2		
As139005	FORWARD	CATGGGGATCCCTTTTCC	60.1	$TTAT_3$	109
	REVERSE	TCCCCATCTTAATACCACTCT	55.3		
As141357	FORWARD	ATACTTCACCGTAGGCTTCA	55.0	CT_4	118
	REVERSE	AGAGAAGGAGTAGCGGATGT	55.6		
As156857	FORWARD	AATCTCCAAATTAATCAGTGC	53.6	$CCTT_3$	105
	REVERSE	GAAGAAGATTTTGTGAACGTG	55.0		
As166543	FORWARD	GCAGAAAAGGCTGTTTTATACT	54.8	$CTAT_3$	108
	REVERSE	AGGCCTTATGGACTAAATACC	54.2		
As166729a	FORWARD	GGAGTATCGGCCTTAACAGTA	56.1	AT_4	105
	REVERSE	AACTCATCAAAAGAGGCATTT	55.6		
As166729b	FORWARD	GAGTATCGGCCTTAACAGTAA	53.9	AG_4	104
	REVERSE	AACTCATCAAAAGAGGCATTT	55.6		
As167143	FORWARD	TGGCAAATATTCCACTGTTAT	54.8	TC_4	110

	REVERSE	TTTTTATGGATGCTGAGAG	51.3		
As171417	FORWARD	TCTACCAACTTCACTTCATCTG	54.6	CA_4	101
	REVERSE	GAAGAAAATCAAAGCTACAAGC	55.3		
As173216	FORWARD	CGTCGTGAGATGGAAGAA	55.5	GT_4	115
	REVERSE	ACAAGGAGAATTGGATGAACT	55.3		
As175951	FORWARD	AGTCTTAAGAAACTGGGGAAG	54.4	TG_4	100
	REVERSE	CGTTGGTCCACTCTCTTACTA	54.6		
As184386	FORWARD	CACAAATAGAGTCGAGGATTG	55.0	TA_4	113
	REVERSE	CAAAAGGAAAAAGTAGACAGTG	53.6		
As188796	FORWARD	TGTAATTCCATGTTCTGAGGA	55.7	GT_4	140
	REVERSE	CTTCAGACTACCAAAGTGGAA	54.6		
As195934	FORWARD	ACCCTTGAGTCACTTGGTTA	54.7	TG_4	102
	REVERSE	ACTCATGCCTTGAACAACA	55.0		
As208556	FORWARD	AACGGATGTAATAATGGGATT	55.1	AT_4	102
	REVERSE	ATTTAGAATGTGAGTGGTGAC	51.6		
As213427	FORWARD	TGACCCGATAGACACTTTTTA	55.0	TC_4	121
	REVERSE	ATGGTGTTAATGACCATTGTG	55.7		
As213687	FORWARD	ATTTGATACCTCGGCTTTATC	55.1	CT_4	100
	REVERSE	ATCATCTCACCCAACATAACT	53.5		
As228706	FORWARD	GGTTGTATTTTCTCATTGAA	50.2	TA_4	107
	REVERSE	CCATTTACATTTCCTAGATTTACC	55.3		
As250590	FORWARD	TCACTTTCCCTAAATAAACGTAA	55.1	TC_4	104
	REVERSE	ATAAACTATCCGGTCTCATCC	54.8		
As272595	FORWARD	TGTTGACAATCTGATCCACTT	55.6	AG_4	101
	REVERSE	GAGGCTTGTCAGTTTGAAAT	54.5		
As279352	FORWARD	ATACGAAAACCTTGACATTAGG	55.2	AG_4	100
	REVERSE	ACAATTGCAGATGTAATGGAC	55.1		
As309843	FORWARD	TCCAACTTTATTGTTTCTGGA	55.0	TC_4	115
	REVERSE	GGGAACCCAACTTTGATACT	55.6		
As316336	FORWARD	CCTCTCGTTTGTTGAATGATA	55.4	AG_4	144
	REVERSE	GTGCAGCAACACAAAGTTTA	55.0		
